# Transcriptomic analysis of the liver in aged laying hens with different intensity of brown eggshell color

**DOI:** 10.5713/ajas.20.0237

**Published:** 2020-10-13

**Authors:** Gi Ppeum Han, Jun-Mo Kim, Hwan Ku Kang, Dong Yong Kil

**Affiliations:** 1 Department of Animal Science and Technology, Chung-Ang University, Anseong 17546, Korea; 2 Poultry Research Institute, National Institute of Animal Science, Rural Development Administration, Pyeongchang 25342, Korea

**Keywords:** Aged Laying Hen, Eggshell Color, Liver, Transcriptome

## Abstract

**Objective:**

Eggshell color is an important indicator of egg quality for consumers, especially for brown eggs. Various factors related to laying hens and their environment affect brown eggshell coloration. However, there have been no studies investigating hepatic functions of laying hens with variable intensity of brown eggshell color. Therefore, this study was aimed to identify potential factors affecting brown eggshell coloration in aged laying hens at the hepatic transcriptomic level.

**Methods:**

Five hundred 92-wk-old Hy-line Brown laying hens were screened to select laying hens with different intensity of brown eggshell color based on eggshell color fans. Based on eggshell color scores, hens with dark brown eggshells (DBE; eggshell color fan score = 14.8) and hens with light brown eggshells (LBE; eggshell color fan score = 9.7) were finally selected for the liver sampling. We performed RNA-seq analysis using the liver samples through the paired-end sequencing libraries. Differentially expressed genes (DEGs) profiling was carried out to identify their biological meaning by bioinformatics.

**Results:**

A total of 290 DEGs were identified with 196 being up-regulated and 94 being down-regulated in DBE groups as compared to LBE groups. The Kyoto encyclopedia of genes and genomes (KEGG) analysis revealed that these DEGs belong to several biological pathways including herpes simplex infection (toll-like receptor 3 [*TLR3*], cyclin-dependent kinase 1, etc.) and influenza A (*TLR3*, radical S-adenosyl methionine domain containing 2, myxovirus [influenza virus] resistance 1, etc.). Genes related to stress response (ceremide kinase like) and nutrient metabolism (phosphoenolpyruvate carboxy-kinase 1, methylmalonic aciduria [cobalamin deficiency] cblB type, glycine receptor alpha 2, solute carrier family 7 member 11, etc.) were also identified to be differentially expressed.

**Conclusion:**

The current results provide new insights regarding hepatic molecular functions related to different intensity of brown eggshell color in aged laying hens. These insights will contribute to future studies aiming to optimize brown eggshell coloration in aged laying hens.

## INTRODUCTION

Eggshell color is one of important indicators of egg quality for consumers, especially in the countries that primarily produce brown eggs. As laying hens are aged, brown color of eggshells becomes faded, which leads to a decrease in consumer preference to brown eggs [1]. Therefore, the production of uniformly dark-brown eggshells has been received substantial attention from layer producers. There have been many efforts to uncover critical factors causing impaired brown eggshell coloration, especially for decreased intensity of brown color, and to find possible treatments for eggshell color problems [2]. A well-known factor responsible for decreased intensity of brown coloration is the aging of laying hens. The primary reason for this phenomenon is believed to be that increasing egg size is accompanied with decreasing or similar production of eggshell colorants in the uterus of laying hens [3]. Although clear mechanisms are not yet known, other factors such as diseases, nutrition, genetics, environment, and stress may also play a role in determining the intensity of brown eggshell color [2]. More importantly, it is likely that these factors are highly interactive in affecting brown eggshell coloration [4]. However, a lack of information for potential factors affecting the intensity of brown eggshell coloration is available, which limits the development of possible treatments for improving brown eggshell coloration in laying hens.

With the recent advance in the next-generation sequencing technique, RNA sequencing (RNA-seq) can profile overall gene expression changes (i.e., transcriptome) among individuals with different phenotypes, which can improve the current knowledge at the molecular level [5]. For poultry, the RNA-seq techniques have been adopted to compare the overall transcriptomic alteration in the liver of broiler chickens exposed to different temperature stress (e.g. heat stress or cold stress) [6,7] and laying hens at different physiological or production stages [8]. Regarding eggshell quality, previous studies have focused primarily on transcriptomic analysis in the uterus, which can reveal the molecular mechanisms that may cause laying hens with different eggshell quality [9,10]. For the eggshell color, Wang et al [11] conducted transcriptomic analysis to identify molecular factors affecting blue coloration in the uterus of duck with blue-green eggshells and reported a large difference in cholesterol biosynthesis and ion levels involved in the modulation of the transporting activity of bile pigment transporters. In laying hens, most of previous experiments have studied protoporphyrin IX (PP9) synthesis and transport to eggshells, specifically in the uterus because PP9 is the main pigment of brown color in eggshells and its synthesis and deposition occur in the uterine [12,13]. However, there has been a lack of data for additional factors affecting brown coloration in eggshells other than PP9 synthesis and deposition in the uterus. The liver is the central organ regulating nutrient metabolism, hormone production, detoxification, decomposition of red blood cells, and immune systems [14], which are considered to potentially affect brown eggshell coloration, although the liver is not the main organ for PP9 synthesis [13,15]. However, to our knowledge, there are no studies that have conducted a transcriptomic analysis to search for novel genes and metabolic pathways in the liver of laying hens with varying the intensity of brown eggshell color.

The aim of this study was to identify potential factors affecting the intensity of brown eggshell color in aged laying hens at the hepatic transcriptomic level.

## MATERIALS AND METHODS

### Animals and sample collection

**Figure 1:**
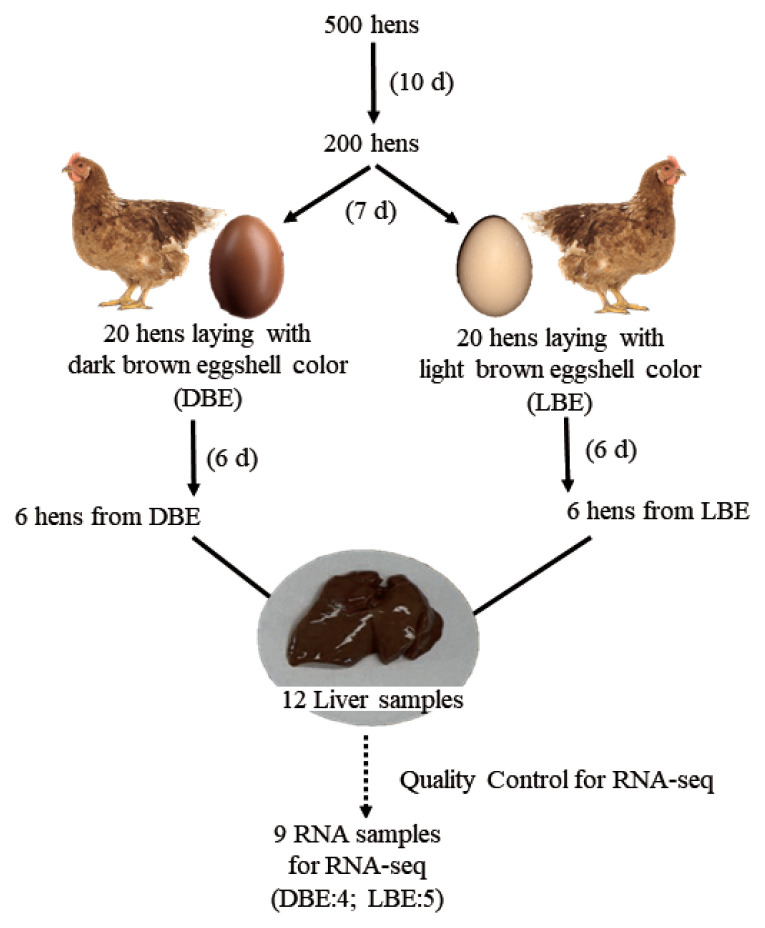
The diagram of the experimental strategy for RNA-Seq. The day in the blank means the recording period of eggshell color. Six hens from each group were selected for RNA-Seq. However, during the quality control process, 2 samples from dark brown eggshell (DBE) group and 1 sample from light brown eggshell (LBE) group were removed, and thus, were not subject to RNA-seq analysis.

All experimental procedures were reviewed and approved by the Animal Care and the Use Committee at Chung-Ang University (IACUC:2018-00002). A total of five hundred 92-wk-old Hy-Line Brown laying hens were used to investigate the eggshell color at the start of the current experiment. All hens were raised in individual cages and the eggshell color of all eggs produced for 10 d was scored based on an eggshell color fan (Samyangsa, Kangwon-do, Korea). After investigation of eggshell color, a total of two hundred hens (100 laying hens with dark brown eggshells [DBE] and 100 laying hens with light brown eggshells [LBE]) were selected. All eggs from the 200 laying hens were continuously reexamined for eggshell color based on an eggshell color fan and a Commission Internationale de l’Eclairage (CIE) L*a*b* color system using a colorimeter (CR-10, Konica Minolta, Tokyo, Japan), and a total of 40 hens (20 laying hens with DBEs and 20 laying hens with LBEs) were selected again. Within each group of eggshell color, 6 hens were finally selected based on additional 6-d measurements of the eggshell color fan and CIE L*a*b* color system and were assigned to DBE and LBE group. In addition to eggshell color, eggshell strength of eggs from DBE and LBE groups was also measured using the Texture analyzer (model TAHDi 500, Stable Micro System, Godalming, UK). All hens were euthanized by CO_2_ asphyxiation at 6 h after their oviposition. The liver was rapidly harvested and immediately frozen in liquid nitrogen for RNA-Seq. All liver samples were stored at −80°C until RNA extraction procedure was performed. The detailed procedure of hen selection and tissue sampling is presented in Figure 1.

### RNA extraction, library construction, and sequencing

Total liver RNA was extracted using TRIzol reagent (Invitrogen, Carlsbad, CA, USA) after grinding the frozen liver sample under liquid nitrogen condition. A total of 12 RNA integrities were checked using an Agilent Technologies 2100 Bioanalyzer and RiboGreen dye (Invitrogen, USA), and quantified using a Trinean DropSense96 spectrophotometer (Trinean, Gentbrugge, Belgium). A quality control criteria set as the ratio of 28S:18S >1 and RNA integrity number >7. RNA samples deviated from the criteria were excluded from fox RNA-Seq analysis. As a result, 9 out of 12 RNAs were passed and subjected to RNA sequencing (DBE, 4 samples; LBE, 5 samples).

RNA-Seq libraries were prepared from total RNA using the TruSeq Stranded mRNA Sample Preparation Kit (Illumina, San Diego, CA, USA). PolyA+ RNA was purified from total RNA using AMPure XP beads and subsequently fragmented to 200 to 700 bp (average 350 bp).

A total of 9 cDNA was synthesized using reverse transcriptase (SuperScript II, Invitrogen, USA) and random primers. The dUTP second-strand marking method was used for the strand-specific RNA-Seq library preparation. Double-stranded DNA was used for library preparation; dsDNA was ligated to the barcoded Truseq adapters. Library amplification was performed by polymerase chain reaction (PCR) on the size selected fragments. The library was sequenced on an Illumina Nextseq 500 sequencer (Illumina, USA) using a paired-end run (2×75 bases).

### Expression quantification of RNA-seq data

The raw reads were trimmed by filtering out adaptor-only nucleotides with 75-bp minimum length, using Trimmomatic (ver 0.36) [16]. Trimmed reads were aligned to the reference genome (galGal4) using HISAT software (ver 2-2.1.0) [17]. To compare reads containing the strand information to those without available standard information, reads were also aligned without using the ‘--rna-strandness RF’ option. We quantified the mapped reads using FeatureCounts [18] with an annotation file (.GTF) for protein coding genes from the Ensembl database [19].

### Differential expressed gene analysis

Differentially expressed genes (DEGs) were identified using the edgeR Bioconductor package based on the generalized linear model, which is used for the analysis of RNA-Seq data by considering gene expression as a negative binomial [20]. The edgeR-robust method was used to reduce the effect of outlier genes [21]. Significance cutoff for differentially expressed genes was set at p-value <0.01.

### Gene ontology and Kyoto encyclopedia of genes and genomes pathway analysis

A gene ontology (GO)-based enrichment (biological process, cellular component, and molecular function) was conducted using the Fisher’s exact test. A functional enrichment analysis was performed using the Database for Annotation, Visualization and Integrated Discovery (DAVID; ver 6.8) [22]. Functional enrichment of the DEGs was also analyzed using DAVID to identify Kyoto encyclopedia of genes and genomes (KEGG) pathways.

### Protein–protein interaction analysis of differential expressed genes

Protein–protein interaction (PPI) analysis of DEGs was based on the Search Tool for the Retrieval of Interacting Genes (STRING) app in cytoscape (ver 3.8.0; http://www.cytoscape.org/) for network analysis. All DEGs were mapped to STRING. A high confidence score of more than 0.7 was considered significant and then disconnected nodes were finally removed.

### Quantitative real-time polymerase chain reaction analysis

To ensure the accuracy of differential expression data obtained from the RNA-Seq, quantitative real-time (qRT)-PCR was carried out for 9 selected DEGs in the liver RNA samples used for RNA-Seq. The total RNA was used for cDNA synthesis using RevertAid First Strand cDNA Synthesis Kit (Thermo scientific, Waltham, MA, USA) according to the standard procedure.

All primer sequences were designed using Primer-BLAST in the NCBI and are listed in (Supplementary Table S1). To confirm the specificity of the primer to a target gene, PCR amplification was performed by modifying the protocol described by Aznar and Alarcon [23]. Briefly, PCR amplification was conducted using cDNA samples and Dream Taq green PCR Master mix (Thermo scientific, USA). Amplification conditions were: an initial cycle at 95°C for 5 min followed by 35 cycles at 95°C for 30 s, annealing temperature for 30 s, 72°C for 1 min, and a final 5-min extension at 72°C. Reactions were carried out in a MyCycler Thermal Cycler System (Bio-rad, Hercules, CA, USA). To conduct RT-PCR, diluted 1 μL cDNA (100 ng) was added to a reaction mix with 10 μL AMPIGENE qPCR Green Mix Lo-ROX (Enzo life science, Farmingdale, NY, USA), 1 μL of each forward and reverse primer (10 pmole), and nuclease-free water was added to obtain a total volume of 20 μL. The RT-PCR was performed using a CFX connect real-time PCR Detection system (Bio-rad, USA). The cycling conditions were as follows: 95°C for 2 min, followed by 40 cycles of amplification (95°C for 5 s and 60°C for 25 s). The relative quantification of gene expression was calculated using 2^−ΔΔ^*^Ct^* method, with glyceraldehyde-3-phosphate dehydrogenase as the reference gene.

## RESULTS

### Comparison of eggshell quality between the DBE and LBE group

As we expected, the DBE group had darker brown eggshells with a greater (p<0.01) eggshell color fan score and CIE a* (redness) value but less (p<0.01) CIE L* (lightness) value than the LBE group (Table 1). However, no differences in eggshell strength and CIE b* (yellowness) value were observed between the DBE and LBE group. These results indicate that the selection of hens in the current experiment was successful.

### Overall assessment for mapping statistics

The paired-end sequence reads were produced with ranges from 17,871,458 to 20,950,555 by 75-bp per sample following the average number being: the DBE group (18,855,063) and LBE group (19,217,252). The adaptor sequence was removed from the total reads and filtering of reads with a low-quality score (>Q30) was performed; DBE group (94.8%) and LBE group (94.9%). More than 87.31% of reads per sample were trimmed. HISAT alignment percentage of all samples is more than 94.97% of clean reads per sample. The detailed alignment statistics and data quality of reads for liver RNA samples are presented in Table 2.

### Differentially expressed genes between DBE and LBE group

A total of 290 genes were differentially expressed in the liver between the DBE and LBE group. Among these DEGs, 196 genes were up-regulated and the other 94 genes were down-regulated in the DBE group. The details of those DEGs identified between the DBE and LBE group are provided in Supplementary Table S2.

**Figure 2:**
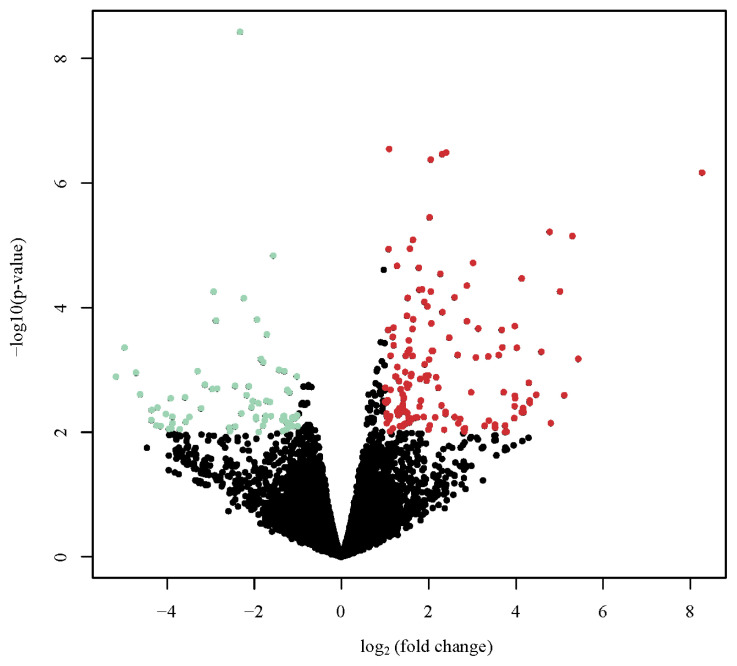
Volcano plot appearing p-values against fold changes. The volcano plot reports log (p-value) for genes as Y variables plotted against their respective log_2_ (fold change) as X variables. The red and green dots indicated significantly up- and down-regulated in dark brown eggshell (DBE) group vs. light brown eggshell (LBE) group, respectively (p<0.01, |log_2_ fold change| >2). The black dots represent no differential expressed genes (DEGs).

Volcano plots of the DEGs visualized their separate profiles between the DBE and LBE group (Figure 2). The top 20 DEGs (10 up-regulated and 10 down-regulated genes) are listed in Table 3.

### Validation of differential expressed gene in the RNA-seq

**Figure 3:**
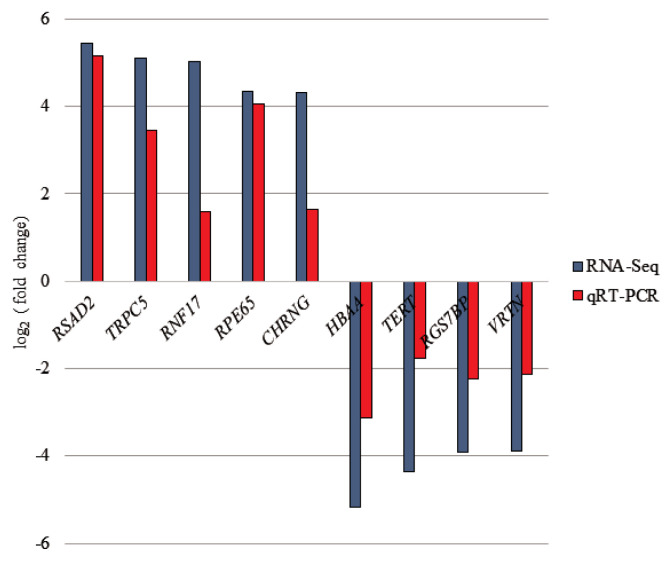
Illustrating of qRT-PCR validation for selected differentially expressed genes (DEGs). The selected genes were placed in X-axis and Y-axis represents log_2_ (fold change) from qRT-PCR and RNA-seq. qRT-PCR, quantitative real-time polymerase chain reaction.

To confirm the credibility of our RNA-Seq results, 9 DEGs were selected for qRT-PCR assay using the same RNA samples as those used for RNA-seq. The expression patterns of 9 DEGs obtained from qRT-PCR were concordant with those obtained from RNA-Seq (Figure 3).

### Gene ontology and Kyoto encyclopedia of genes and genomes pathway analysis of the differential expressed genes

**Figure 4:**
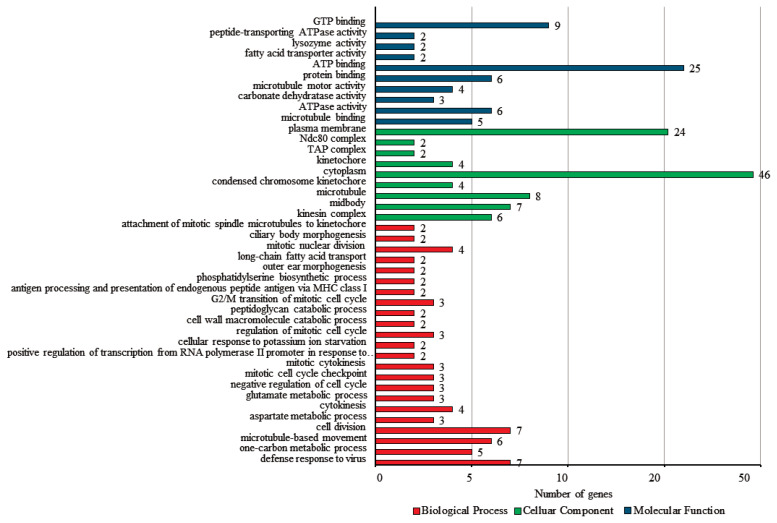
Gene ontology (GO) classification of differential expressed genes (DEG).

To verify the biological functions of the DEGs, the GO and KEGG analyses were conducted. Among 290 DEGs, a total of 241 genes were classified in the three categories of GO analysis, with 30.7% (74) classified as biological process, 42.7% (103) classified as cellular components, and 26.6% (64) classified as molecular function (Figure 4). For the biological process group, defense response to virus (9.5%, gene count; 7) and cell division (9.5%, gene count; 7) showed slightly higher clusters. Within the cellular components, cytoplasm (44.7%, gene count; 46) was the most prominent group, followed by plasma membrane (23.3%, gene count; 24). ATP binding (39.1%, gene count; 25) was the most enriched in the molecular function.

According to the KEGG analysis, a total of 176 genes were associated with 80 pathways (Supplementary Table S3). The enriched pathways included metabolic pathways, herpes simplex infection, influenza A, cell cycle, apelin signaling pathway, ubiquitin mediated proteolysis, neuroactive ligand-receptor interaction, cell adhesion molecules, tight junction, cellular senescence, FoxO signaling pathway, glycerophospholipid metabolism, peroxisome proliferator activated receptor (PPAR) signaling pathway, etc. (Table 4).

### Protein–protein interaction analysis of the differential expressed genes

**Figure 5:**
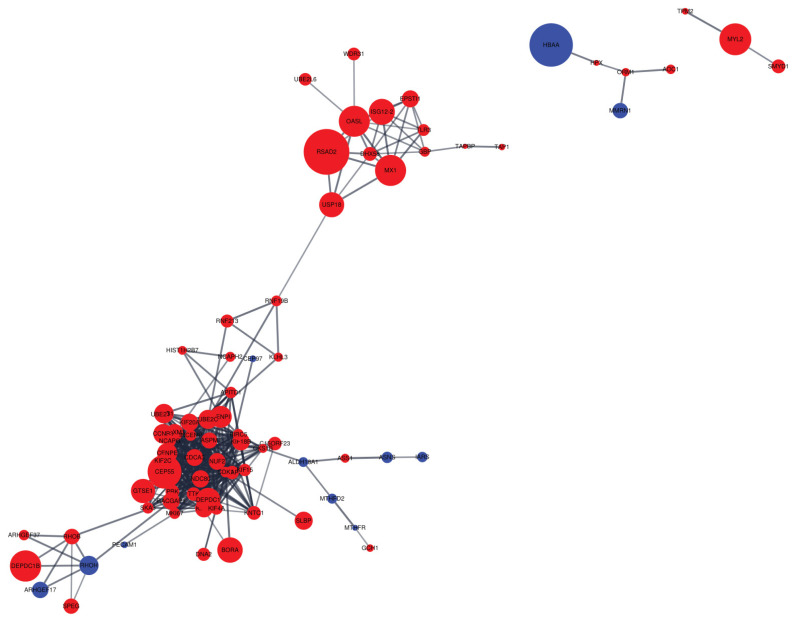
Protein-protein interaction (PPI) network for differentially expressed genes (DEGs). The red node represents the up-regulated gene, whereas the blue node represents the down-regulated gene. All edges represent protein-protein interaction. The node size indicates |log_2_ fold change|.

Cytoscape software was employed to visualize PPI networks of DEGs (Figure 5). A total of 231 nodes and 426 edges were incorporated, which resulted in an average node degree of 6.68 and average local clustering coefficient of 0.432 (Supplementary Table S4).

## DISCUSSION

The liver is one of the most important organs in the body and carries out a variety of critical functions involved in nutrient metabolism such as carbohydrate, protein and lipid metabolism [14]. The liver also plays a role in regulating hormonal responses, detoxification, and immune systems [14]. Therefore, the brown coloration and its intensity of eggshells may also be influenced by changes in liver functions. However, researches on altered hepatic molecular functions of laying hens in relation to brown eggshell color are scarce.

As expected, the eggshell color was significantly different between selected groups for laying hens with DBE and LBE (eggshell color fan score = 14.8 vs 9.7 for DBE and LBE, respectively). Therefore, we confirmed that the selected groups offered trustworthy data for transcriptomic analysis of the liver of laying hens with different intensity of brown eggshell color.

The KEGG analysis predicted that 176 DEGs were involved in 80 pathways. Genes related to herpes simplex infection and influenza A were highly enriched. Many studies have reported that viral diseases are associated with a decrease in the intensity of brown eggshell color, indicating that the prevention of viral diseases may prevent impairments in brown eggshell color [24]. It was also noted that PPAR signaling pathway was found to be enriched in DEGs between DBE and LBE. PPAR signaling pathway is related to lipid metabolism [25]. DEGs (phosphoenolpyruvate carboxykinase 1 [*PCK1*], solute carrier family 27 member 1, FP325317.1) involved in PPAR signaling pathways have been proven to be functional in lipid metabolism and these DEGs were up-regulated. Previous studies have reported that up-regulation of three genes involved in the PPAR signaling pathway is associated with decreased lipogenesis [25–27]. Indeed, GO analysis revealed that all DEGs in ‘defense response to virus’ category, except glycerol-3-phosphate acyltransferase, were up-regulated in laying hens with DBE as compared to laying hens with LBE. It is suggested, therefore, that the defense response of laying hens against viral diseases is a critical function that affects brown eggshell coloration of laying hens. Moreover, several DEGs associated with immune responses to viral infection, stress response, and nutrient metabolism were also identified in the current study.

The PPI networks provide opportunities to further research the relationship between DEGs through conserved pathways and protein complexes [28]. We found that up-regulated genes were more clustered than down-regulated genes, indicating a network of up-regulated genes in the liver appears to have a stronger effect on the intensity of eggshell brownness. We discovered that the second largest hub included various nodes associated with immune responses (e.g., herpes simplex infection and influenza A), which is close to the upper KEGG pathway observed in the current experiment. The two proteins with the most interactions are the assembly factor for spindle microtubules (*ASPM*) and kinesin family member 20A (*KIF20A*). These two proteins account for approximately 10% of the total interactions, which means that these two proteins may contribute considerably to the difference in brown eggshell coloration. Both proteins are known to play a role in cell mitosis and cell proliferation [29, 30]. Thus, our observation that *ASPM* and *KIF20A* expressions were significantly increased in the DBE group than in the LBE group may indicate that DBE group had a better cell proliferation capacity than LBE group. Previous study also demonstrated that the forkhead box M1, which is commonly interacted with the two proteins, is associated with liver regeneration and tissue repair in mouse [31]. Thus, we speculate that DBE group may exhibit a higher ability of liver regeneration and repair as compared to LBE group. However, this speculation should be validated in the future studies.

### Up-regulated genes in laying hens with DBE

Radical S-adenosyl methionine domain containing 2 (*RSAD2*) is a member of the interferon (IFN)-stimulated genes (ISGs) that is involved in the innate immunity [32]. Jang et al [33] reported that mature dendritic cell functions were markedly attenuated in *RSAD2*-knockdown mice. Moreover, *RSAD2* knockdown was related to a decreased ability to promote proinflammatory cytokine production and induce T cell proliferation. In this study, *RSAD2* expression in the liver was up-regulated with a log_2_ fold change of 5.44 in laying hens with DBE than those with LBE. Therefore, it is speculated that up-regulation of *RSAD2* may represent improved immune responses, and therefore, alterations in immune systems may be one factor affecting the intensity of brown eggshell color.

Ceremide kinase like (*CERKL*) is a gene homology with ceramide kinase (*CERK*), which is an enzyme belonging to the family of transferases [34]. Diseases related to *CERK* include retinitis pigmentosa disease, a retinal disease causing degeneration of photoreceptors in humans [35]. Previous research reported that *CERKL*-knockdown zebrafish showed a retinal degeneration [36]. However, overexpression of *CERKL* was reported to show a protective effect against oxidative stress induced by a high lighting in mice [34]. Therefore, *CERKL* is likely responsible for the stress responses and protection of photoreceptor cells. In the present study, the expression of *CERKL* was increased in laying hens with DBE than those with LBE, with a log_2_ fold change of 4.59, indicating that this gene may play a role in brown eggshell coloration. However, no previous studies have investigated the relationship between *CERKL* expression and eggshell coloration in poultry.

Retinal pigment epithelium-specific protein 65 kDa (*RPE65*) is an important isomerase associated with retinal pigmentation in humans [37]. This gene was up-regulated with a log_2_ fold change of 4.33 in laying hens with DBE than those with LBE in this study. Previous experiment indicated that *RPE65*-deficient patients have shown early loss of ocular photoreceptors [38]. Based on the results for *CERKL* and *RPE65*, it may be hypothesized that retinal metabolism in the liver of laying hens may be associated with brown eggshell coloration. However, it remains unclear how the up-regulation of *CERKL* and *RPE65* leads to an increase in the intensity of brown eggshell coloration in aged laying hens.

Myxovirus (influenza virus) resistance 1 (*MX1*) gene also belongs to ISGs which IFN-induced anti-viral effectors in animals [39]. In the current study, *MX1* expression was up-regulated with a log_2_ fold change of 3.69 in the DBE group, which is similar to the result for *RSAD2*. According to Pasick et al [40], the *MX1* gene may be involved in the inhibition of influenza A virus replication by preventing primary viral mRNA synthesis in laying hens. We speculate that the up-regulation of *MX1* expression in the liver may protect laying hens from viral diseases that are responsible for decreasing intensity of brown eggshell color.

The *PCK1* is known to play a role in regulating gluconeogenesis in the liver [41]. The results of the present study indicate that *PCK1* expression was significantly increased in the DBE group than in the LBE group, with a log_2_ fold change of 2.84. The *PCK1* is reported to be essential for regulating lipid metabolism and glucose homeostasis and for preventing insulin resistance in mice [42]. Zhou et al [25] showed that the *PCK1* was up-regulated in laying hens fed dietary alfalfa saponin extract that has been reported to have cholesterol lowering effects. Therefore, we speculate that there may be a difference in energy metabolism and insulin resistance between the DBE and LBE groups, indicating that laying hens with DBE have a greater ability to regulate nutrient metabolism, especially for glucose, than laying hens with LBE.

Methylmalonic aciduria (cobalamin deficiency) cblB type (*MMAB*) encodes a protein that catalyzes the final step in the conversion of vitamin B_12_ into adenosylcobalamin [43]. In this study, *MMAB* expression in the liver was greater by a log_2_ fold change of 2.73 in the DBE group than in the LBE group. Willer et al [44] reported that *MMAB* was associated with lipid metabolism, sharing a common promotor with sterol regulatory element-binding protein 2, which is known to regulate cholesterol and fatty acid synthesis. *MMAB* also participates in a metabolic pathway for cholesterol degradation. Previous study has reported that non-alcoholic steatohepatitis (NASH) in mouse models showed a down-regulation of *MMAB* [45]. Therefore, we may suggest that the brown eggshell coloration in laying hens is also related to hepatic lipid metabolism and, furthermore, that laying hens with LBE may show abnormal lipid metabolism such as fatty liver diseases.

Solute carrier family 25, member 15 (*SLC25A15*) encodes a mitochondrial carrier protein that catalyzes the electroneutral exchange of ornithine for H^+^ and functions as an ammonium detoxification [46]. Ammonia is a major factor that negatively affect laying performance and health in poultry. Long-term exposure to ammonia can cause severe hepatic injury [47]. In our study, the *SLC25A15* gene was up-regulated by a log_2_ fold change of 1.41 in laying hens with DBE than in those with LBE. Thus, we can hypothesize that up-regulation of *SLC25A15* could enhance the ability to detoxify ammonia in the liver, although it is difficult to find a direct evidence for a link of upregulation of *SLC25A15* to the production of darker brown eggshells.

Toll-like receptor (TLR) family plays a fundamental role in pathogen recognition and activation of the innate immunity [48]. The expression of *TLR3* has been related to increased protection against Marek’s disease virus in chicken embryo fibroblasts [49]. This gene was up-regulated with a log_2_ fold change of 1.41 in laying hens with DBE than those with LBE in this study. It is likely, therefore, that the elevated expression of *TLR3* may contribute to an improvement in the host defense against viral disease.

### Down-regulated genes in laying hens with DBE

The major pigment in brown eggshells is PP9 [1]. Moreover, the PP9 is an immediate precursor of heme synthesis [1]. Previous researchers have demonstrated that PP9 is synthesized in the shell gland, where it is secreted and deposited into eggshell layers [50]. However, some authors have also reported that PP9 can be derived from spent red blood cells through the degradation and metabolism of hemoglobin in the liver [13]. In the present study, Gallus gallus hemoglobin, alpha 1 (*HBAA*) expression in the liver was down-regulated by a log_2_ fold change of 5.17 in laying hens with DBE than those with LBE. In our previous study of uterine transcriptomic analysis of laying hens with different intensity of brown eggshell color (i.e., DBE vs LBE), however, the expression pattern was contrary to the current results, demonstrating that the expression of hemoglobin-related gene was significantly up-regulated in the uterus of hens with DBE than hens with LBE (data are not published). It was reported that decreased PP9 synthesis was correlated with increased heme synthesis in the uterus of laying hens with LBEs [15]. Therefore, we hypothesize that the down-regulation of *HBAA* in the liver might be related to up-regulation of PP9 synthesis in the uterus where the pigment of eggshell coloration is primarily synthesized; however, this hypothesis should be tested in the further studies. In addition, the previous experiment investigated several genes related to heme metabolism in the liver and reported that hepatic expression of aminolevulinic acid synthase-1, which is the rate-limiting enzyme for heme synthesis and subsequently PP9 synthesis, was increased in DBE group as compared to LBE group [12]. However, we failed to detect any change in the gene expression as reported by Li et al [12]. The reason may be the different sensitivity to detect gene expressions between RNA-seq used in this experiment and RT-PCR used in Li et al [12].

Glycine receptor alpha 2 (*GLRA2*) is a subunit of the glycine receptors, which are ligand-gated chloride channels induced by taurine, β-alanine, and glycine [51]. It has been documented that glycine receptors are associated with regulation of inhibitory neurotransmission in the spinal cord and brain [52]. In this study, *GLRA2* expression in the liver was down-regulated by a log_2_ fold change of 4.02 in the DBE than in the LBE. Based on the previous study, laying hens with DBE may show less efficient neurotransmission than those with LBE groups. However, it was reported that *GLRA2* knockout mice showed little molecular alterations in the nervous system related to glycine-mediated neurotransmission [53]. There have been no studies related to glycine receptors in poultry. Therefore, it is not clear why laying hens with DBE exhibited a large reduction in hepatic *GLRA2* expression as compared to those with LBE. Further studies on *GLRA2* functions in the liver are necessary for elucidating its role in brown eggshell coloration of laying hens.

Solute carrier family 7 member 11 (*SLC7A11*) is the functional light chain subunit of the cystine/glutamate antiporter system and participates in the cystine uptake specificity [54]. *SLC7A11* has also been associated with cell death regulated by reactive oxygen species in human cancers [55]. In NASH, the expression of *SLC7A11* was reported to be up-regulated in human hepatoma cells with a positive correlation to the expression of lipid-associated genes [56]. The results of the present study indicated that the expression of *SLC7A11* was down-regulated by a log_2_ fold change of 3.57 in laying hens with DBE than in laying hens with LBE. Based on the results for some DEGs (*MMAB* and *SLC7A11*), lipid metabolism in the liver of laying hens may be associated with brown eggshell coloration.

It should be noted that it will be better to have uterine transcriptomic data and analyze the correlation between the liver and uterine to understand more detailed molecular mechanisms for brown eggshell colorations in aged laying hens. Moreover, additional data for health status and immune responses of aged laying hens with different eggshell color (DBE vs LBE group) are likely required to support our findings of various hepatic genes differentially expressed in the current experiment; however, we did not obtain additional measurements. The further studies are required to overcome the limitation in the current experiment.

## CONCLUSION

This study conducted a comparative transcriptomic analysis in the liver of aged laying hens with different intensity of brown eggshell color. We found that several genes related to immune responses and anti-viral action were up-regulated in the liver of aged laying hens with DBE. Expression of several genes related to lipid metabolism was also changed in aged laying hens with different intensity of brown eggshell color. These results provide new insights regarding hepatic molecular mechanisms in aged laying hens with variable intensity of brown eggshell color, which may aid in the development of possible treatments aimed to optimize brown eggshell color in aged laying hens.

## Figures and Tables

**Table 1:** Eggshell color and strength in laying hens with either dark brown eggshells or light brown eggshells

Items	Group		SEM	p-value

	DBE	LBE		
Eggshell color (color fan)	14.8	9.7	0.31	<0.01
Eggshell color (CIE values)				
L*	49.0	62.3	0.79	<0.01
a*	24.0	16.6	0.68	<0.01
b*	25.5	24.9	1.36	0.75
Eggshell strength (kg/cm^2^)	3.49	3.46	0.282	0.96

DBE, dark brown eggshell; LBE, light brown eggshell; SEM, standard error of the mean; CIE, Commission Internationale de l’Eclairage.

**Table 2:** Summary for sequence quality and alignment information of the liver samples

Items	D1	D2	D3	D4	L1	L2	L3	L4	L5
Group	DBE	DBE	DBE	DBE	LBE	LBE	LBE	LBE	LBE
Raw read count	19,491,608	17,871,458	19,275,833	18,781,352	20,950,555	17,971,645	18,028,445	18,621,472	20,514,142
GC content (%)	47	48	48	48	47	48	47	47	46
Q20 ratio (%)^1)^	99.97	99.99	99.98	99.97	99.99	99.97	99.98	99.98	99.98
Q30 ratio (%)^1)^	95.0	94.2	95.1	94.9	95.1	94.8	95.0	94.7	95.0
Both surviving reads	17,228,714 (88.39%)	15,837,745 (88.62%)	17,160,235 (89.02%)	16,508,234 (87.90%)	18,746,897 (89.48%)	15,691,780 (87.31%)	15,894,913 (88.17%)	16,443,627 (88.30%)	18,135,648 (88.41%)
Overall alignment rate (%)	95.36	95.69	94.97	95.09	95.43	95.36	96.03	95.34	96.23

DBE, dark brown eggshell; LBE, light brown eggshell; GC, guanine-cytosine.

1) Q20 and Q30 represent the proportion of based with a Phred Quality score.

**Table 3:** Detailed information of the top 20 most differentially expressed genes responsible for different brown eggshell color

Ensembl gene ID	Gene symbol	Gene information	Log_2_ fold change (DBE/LBE)	p-value	Up/Down (DBE/LBE)
ENSGALG00000010722	*SERAF*	Schwann cell-specific EGF-like repeat autocrine factor	8.27	<0.01	Up
ENSGALG00000016400	*RSAD2*	Radical S-adenosyl methionine domain containing 2	5.44	<0.01	Up
ENSGALG00000027959	uncharacterized	-	5.30	<0.01	Up
ENSGALG00000007972	*TRPC5*	Transient receptor potential cation channel, subfamily C, member 5	5.11	<0.01	Up
ENSGALG00000017149	*RNF17*	Ring finger protein	5.02	<0.01	Up
ENSGALG00000027989	*SBDSL*	Shwachman-Bodian-Diamond syndrome-like	4.80	0.01	Up
ENSGALG00000007208	*FCGBP*	Fc fragment of IgG binding protein	4.78	<0.01	Up
ENSGALG00000008910	*CERKL*	Ceramide kinase like	4.59	<0.01	Up
ENSGALG00000021729	*MIR193B*	Gga-mir-193b	4.47	<0.01	Up
ENSGALG00000011259	*RPE65*	Retinal pigment epithelium-specific protein 65kDa	4.33	<0.01	Up
ENSGALG00000007468	*HBAA*	Gallus gallus hemoglobin, alpha 1(HBAA), mRNA	−5.17	<0.01	Down
ENSGALG00000011808	*CCR9*	Chemokine (C-C motif) receptor 9	−4.97	<0.01	Down
ENSGALG00000020492	*MUC6*	Mucin 6, oligomeric mucus/gel-forming	−4.71	<0.01	Down
ENSGALG00000018276	*MIR126*	Gga-mir-126	−4.61	<0.01	Down
ENSGALG00000005762	uncharacterized	.	−4.35	0.01	Down
ENSGALG00000013183	*TERT*	Telomerase reverse transcriptase	−4.35	<0.01	Down
ENSGALG00000025564	uncharacterized	-	−4.24	0.01	Down
ENSGALG00000004128	*CYSLTR1*	Cysteinyl leukotriene receptor 1	−4.22	<0.01	Down
ENSGALG00000015276	uncharacterized	-	−4.14	0.01	Down
ENSGALG00000016571	G*LRA2*	Glycine receptor, alpha 2	−4.02	0.01	Down

DBE, dark brown eggshell; LBE, light brown eggshell.

**Table 4:** Kyoto encyclopedia of genes and genomes pathways enriched with differentially expressed genes responsible for different brown eggshell color

Pathway	Pathway definition	The number of gene	Genes
path:gga01100	Metabolic pathways	24	*MTHFR, MTHFD2, ASNS, LTC4S, GCH1, ST3GAL1, GCLC, AKR1B1L, GPAM, MMAB, MAOA, IMPA2, NMRK2, BCMO1, ASS1, PTDSS1, GALNT10, PCK1, PTGDS, ETNPPL, DGUOK, GATM, ALDH18A1*, uncharacterized
path:gga05168	Herpes simplex infection	6	*TLR3, TAP2, TAP1, CDK1*, ENSGALG00000001478, uncharacterized
path:gga05164	Influenza A	5	*TLR3, RSAD2, MX1*, ENSGALG00000001478, uncharacterized
path:gga04110	Cell cycle	5	*PTTG1, TTK, CDKN2B, CCNB3, CDK1*
path:gga04371	Apelin signaling pathway	4	*CTGF, JAG1, MYL2, PRKAG3*
path:gga04120	Ubiquitin mediated proteolysis	4	*UBE2C, SOCS1*, ENSGALG00000001478, uncharacterized
path:gga04080	Neuroactive ligand-receptor interaction	4	*GLRA2, CHRNG, GABRD, CYSLTR1*
path:gga04514	Cell adhesion molecules (CAMs)	4	*CLDN4, NLGN3, LRRC4C, PECAM1*
path:gga04530	Tight junction	4	*CLDN4, MYL2, PRKAG3, A5HUM9_CHICK*
path:gga04218	Cellular senescence	4	*FOXM1, CDKN2B, CCNB3, CDK1*
path:gga04068	FoxO signaling pathway	4	*PCK1, PRKAG3, CDKN2B, CCB3*
path:gga00250	Alanine, aspartate and glutamate metabolism	3	*ASNS, ASS1, DDO*
path:gga00590	Arachidonic acid metabolism	3	*LTC4S, GPX3, PRGDS*
path:gga04261	Adrenergic signaling in cardiomyocytes	3	*SCN5A, TPM2, MYL2*
path:gga04910	Insulin signaling pathway	3	*SOCS1, PCK1, PRKAG3*
path:gga00564	Glycerophospholipid metabolism	3	*GPAM, PTDSS1, ETNPPL*
path:gga00330	Arginine and proline metabolism	3	*MAOA, GATM, ALDH18A1*
path:gga00910	Nitrogen metabolism	3	*CA9, CA13, CA12*
path:gga03320	PPAR signaling pathway	3	*PCK1, SLC27A1*, FP325317.1
