# Supplementary material for: Transcriptomic analysis of the liver in aged laying hens with different intensity of brown eggshell color
Source: Anim Biosci. 2020 Oct 13;34(5):811–23. doi: 10.5713/ajas.20.0237 (PMC8100479; doi:10.5713/ajas.20.0237)
Supplement: Supplementary file 1 [file ajas-20-0237-suppl.pdf]

Table S1.The primers sequences of 9 selected differentially expressed genes and 1 reference gene

Gene	GeneBank	Forward primer(5'-3')	Reverse primer(5'-3')	Product length(bp)	Ta(℃)
RSAD2	NM_001318443.1	GAACGGTGGTTCAAGAAGTA	GCAGATTTTCCACATGGTTC	127	50
TRPC5	XM_420310.6	AAATTGTCAGTCTGGTCTCC	GGAAAAATAAGGCTGGCATC	186	60
RNF17	XM_015279441.2	TAGCCTTTGGTATCGAGGTA	CATGGTATGCAAAATGGAGG	150	60
RPE65	NM_204884.1	CTGAGACAATGTACAGCCAG	CTGTGACGTGAGCAGTG	102	60
CHRNG	NM_001031568.2	CCCCGATAAATACGATGACA	GTACACTAGCACGTTGGTAT	130	60
HBAA	NM_001004376.3	CACTTCGATCTGTCACACG	GGAGTTTGAAGTTGACAGGG	169	60
TERT	NM_001031007.1	GGGATACTATGGAACTGCTG	TCAAGTAGAGATGCTTTCAGT	188	55
RGS7BP	XM_001234191.5	CTGCATAAGGGTTTAGTGGT	ATATTCAGTTGGTGCCACAT	156	60
VRTN	XM_015287674.2	CCTTGTACATGAGGGATCAG	CACACAGTACACTGGTATCC	153	60
GAPDH	NM_204305	TGCTGCCCAGAACATCATCC	ACGGCAGGTCAGGTCAACAA	142	60

Table S2. The details of those DEGs identified between the DBE and LBE group

	Ensembl_Gene_ID	Gene_Symbol	logFC(DBE/LBE)	p-value
1	ENSGALG00000010722		8.274117105	6.81035E-07
2	ENSGALG00000016400	RSAD2	5.436570792	0.000664659
3	ENSGALG00000027959	.	5.299780145	7.09145E-06
4	ENSGALG00000007972	TRPC5	5.113365682	0.002545842
5	ENSGALG00000017149	RNF17	5.017864242	5.50752E-05
6	ENSGALG00000027989	SBDSL	4.804679262	0.007142291
7	ENSGALG00000007208	FCGBP	4.779513306	6.07692E-06
8	ENSGALG00000008910	CERKL	4.59074624	0.000510628
9	ENSGALG00000021729	MIR193B	4.472101627	0.002499102
10	ENSGALG00000011259	RPE65	4.32575321	0.003368593
11	ENSGALG00000007906	CHRNG	4.324201303	0.003102398
12	ENSGALG00000025951	gga-mir-6650	4.301354349	0.001606088
13	ENSGALG00000022335	.	4.17997521	0.004767164
14	ENSGALG00000012051	STAC	4.168785741	0.004100336
15	ENSGALG00000028095	HOXA1	4.140689347	0.00478647
16	ENSGALG00000005245	SGPP2	4.137917863	3.4053E-05
17	ENSGALG00000006639	CEP55	4.025983475	0.000441576
18	ENSGALG00000018244	MIR125B2	3.986411788	0.002861654
19	ENSGALG00000019141		3.978615748	0.00019869
20	ENSGALG00000012053	FAM83F	3.978331281	0.002603208
21	ENSGALG00000014634	PMEL	3.974678189	0.003821204
22	ENSGALG00000018534	FP325317.1	3.968640251	0.005940089
23	ENSGALG00000004582	MYL2	3.791875195	0.009805624
24	ENSGALG00000018374	.	3.790375952	0.009811153
25	ENSGALG00000027569	CLDN4	3.789398276	0.009814775
26	ENSGALG00000026276	TCF15	3.762264684	0.009911009
27	ENSGALG00000001282	GABRD	3.761721439	0.009912564
28	ENSGALG00000006354	GAL3ST2	3.758829649	0.007823598
29	ENSGALG00000024266	.	3.756769314	0.009929925
30	ENSGALG00000014729	DEPDC1B	3.724050325	0.002278938
31	ENSGALG00000016142	MX1	3.692619813	0.00043737
32	ENSGALG00000013723	OASL	3.683025213	0.000227645
33	ENSGALG00000027661	.	3.610968676	0.000576531
34	ENSGALG00000002557	ADGRD1	3.532924422	0.008413416
35	ENSGALG00000015622	EYA1	3.52702653	0.007567589
36	ENSGALG00000011727	.	3.526086923	0.008439457
37	ENSGALG00000002097		3.52483575	0.007574479
38	ENSGALG00000029165	.	3.52341761	0.00757908
39	ENSGALG00000029117	.	3.52341761	0.00757908
40	ENSGALG00000016962		3.384419456	0.006511818
41	ENSGALG00000006384	.	3.37060346	0.00060946
42	ENSGALG00000011360	PRKAG3	3.288102817	0.007896345
43	ENSGALG00000006202	SCNN1B	3.142307841	0.000216668
44	ENSGALG00000013575	IFI6	3.087252162	0.000629846
45	ENSGALG00000016931	BORA	3.021721142	1.92147E-05
46	ENSGALG00000013057	USP18	2.979152303	0.002291705
47	ENSGALG00000011275	DEPDC1	2.884285352	0.000165574
48	ENSGALG00000004897	GTSE1	2.878498756	4.4517E-05
49	ENSGALG00000007636	PCK1	2.839523752	0.008642205
50	ENSGALG00000005862	SBSPON	2.822526552	0.009758227
51	ENSGALG00000024628	.	2.809596141	0.008926324
52	ENSGALG00000003868	MMAB	2.727204187	0.006092281
53	ENSGALG00000013143	PDE3A	2.683608125	0.007217278

54	ENSGALG00000015903	FILIP1	↑	2.670262383	✓	0.000575543
55	ENSGALG00000027704	.	↑	2.60063305	✓	0.005703301
56	ENSGALG00000005038	CENPI	↑	2.599704367	✓	6.83631E-05
57	ENSGALG00000009855	TBC1D9	↑	2.476410422	✓	0.000302807
58	ENSGALG00000013208	CENPE	↑	2.405506535	✓	3.24899E-07
59	ENSGALG00000028982	.	↑	2.391047644	✓	0.00506439
60	ENSGALG00000009639	.	↑	2.389778198	✓	0.004705398
61	ENSGALG00000014759	STYK1	↑	2.360075077	✓	0.009166406
62	ENSGALG00000025810	CCNB3	↑	2.320853563	✓	0.00011819
63	ENSGALG00000001506	PTTG1	↑	2.315435606	✓	3.4282E-07
64	ENSGALG00000004880	SYCP2	↑	2.298484689	✓	0.003713728
65	ENSGALG00000026380	UBE2C	↑	2.273111813	✓	2.86407E-05
66	ENSGALG00000008822	.	↑	2.225802469	✓	0.001929417
67	ENSGALG00000000956	GGCL1	↑	2.174214134	✓	0.001306909
68	ENSGALG00000015712	SLBP	↑	2.15542404	✓	0.005766863
69	ENSGALG00000008095	KIF23	↑	2.102845575	✓	0.000494515
70	ENSGALG00000003231	KIF20A	↑	2.082837841	✓	0.000496279
71	ENSGALG00000000878	KIF18B	↑	2.078883292	✓	0.0004936
72	ENSGALG00000014801	NDC80	↑	2.06735751	✓	0.000179163
73	ENSGALG00000006271	RACGAP1	↑	2.055344817	✓	5.51678E-05
74	ENSGALG00000027864	NFKBIA	↑	2.051256753	✓	4.19727E-07
75	ENSGALG00000016964	EPSTI1	↑	2.030294452	✓	0.007770484
76	ENSGALG00000028830	CCDC42B	↑	2.025931864	✓	3.56045E-06
77	ENSGALG00000014513	CDCA3	↑	2.014234229	✓	0.000680986
78	ENSGALG00000003438	NUF2	↑	2.000117595	✓	0.009069913
79	ENSGALG00000013420	FOXM1	↑	1.99611109	✓	0.001231727
80	ENSGALG00000010122	SH2D4A	↑	1.977658876	✓	9.57388E-05
81	ENSGALG00000026433	.	↑	1.957488943	✓	0.001482609
82	ENSGALG00000027993	MXD3	↑	1.940701196	✓	0.001246973
83	ENSGALG00000011190	PLACL2	↑	1.912364783	✓	0.000814083
84	ENSGALG00000002338	ASPM	↑	1.906913977	✓	8.06025E-05
85	ENSGALG00000000918	CCDC103	↑	1.890085551	✓	0.005696623
86	ENSGALG00000016485	RHOB	↑	1.854271913	✓	5.10448E-05
87	ENSGALG00000028386	SPEG	↑	1.827476633	✓	0.003109345
88	ENSGALG00000008992	ETNPPL	↑	1.8103842	✓	0.001391841
89	ENSGALG00000000761	TSKU	↑	1.794128165	✓	0.003889592
90	ENSGALG00000019864	.	↑	1.791680697	✓	5.15523E-05
91	ENSGALG00000026125	.	↑	1.780552387	✓	2.29203E-05
92	ENSGALG00000000720		↑	1.738428827	✓	0.005778077
93	ENSGALG00000008803	.	↑	1.701314886	✓	0.005835668
94	ENSGALG00000023821	DHX58	↑	1.648223098	✓	0.00015401
95	ENSGALG00000015935	SMYD1	↑	1.643610876	✓	8.1686E-06
96	ENSGALG00000004515	KNTC1	↑	1.636021407	✓	0.000595681
97	ENSGALG00000016582	PBK	↑	1.635271092	✓	0.000219443
98	ENSGALG00000001478		↑	1.61659626	✓	0.006176948
99	ENSGALG00000015874	TTK	↑	1.609779057	✓	0.006086026
100	ENSGALG00000004706	KNSTRN	↑	1.608619006	✓	0.00116961
101	ENSGALG00000017025	CKAP2	↑	1.599915561	✓	0.001258172
102	ENSGALG00000016200	SIK1	↑	1.575280356	✓	1.14042E-05
103	ENSGALG00000012437	IGFBP1	↑	1.563955321	✓	0.000471693
104	ENSGALG00000004037	DNA2	↑	1.559108672	✓	0.00690476
105	ENSGALG00000021340	CA9	↑	1.558939338	✓	0.006999627
106	ENSGALG00000001470	STOML1	↑	1.554870555	✓	0.001738228
107	ENSGALG00000016761	LYG2	↑	1.547455951	✓	0.0003371
108	ENSGALG00000006988	RNF213	↑	1.546024039	✓	0.005905662
109	ENSGALG00000007158	SOCS1	↑	1.524850923	✓	6.97417E-05
110	ENSGALG00000008859	WDR31	↑	1.521670776	✓	0.000523164

111	ENSGALG00000010120	KIF2C	↑	1.516896389	✓	0.004936158
112	ENSGALG00000000102	UBE2T	↑	1.513830318	✓	0.001929286
113	ENSGALG00000029181	UBE2L6	↑	1.504932184	✓	0.000135257
114	ENSGALG00000008635		↑	1.498560656	✓	0.001671142
115	ENSGALG00000004195	KIF4A	↑	1.479787715	✓	0.000592962
116	ENSGALG00000011901	KIF15	↑	1.471998575	✓	0.004659207
117	ENSGALG00000026970		↑	1.470453893	✓	0.007298197
118	ENSGALG00000023626	NTN1	↑	1.465825934	✓	0.001078797
119	ENSGALG00000005961	DGUOK	↑	1.443050026	✓	0.00747613
120	ENSGALG00000014425	NCAPG	↑	1.4374253	✓	0.002785144
121	ENSGALG00000021848	AVDL	↑	1.430188723	✓	0.002905899
122	ENSGALG00000025751	NMRK2	↑	1.413518361	✓	0.003572965
123	ENSGALG00000013468	TLR3	↑	1.408100391	✓	0.0038592
124	ENSGALG00000017032	SLC25A15	↑	1.405652458	✓	0.002472536
125	ENSGALG00000023818	.	↑	1.404662573	✓	0.008064271
126	ENSGALG00000000504	ETV7	↑	1.394967411	✓	0.004483874
127	ENSGALG00000024481	APITD1	↑	1.362899936	✓	0.004805974
128	ENSGALG00000009700	PDK4	↑	1.36287841	✓	0.002042603
129	ENSGALG00000017128	SKA3	↑	1.344870645	✓	0.008004838
130	ENSGALG00000007537	INCENP	↑	1.333419713	✓	0.005045577
131	ENSGALG00000016546	RBBP7	↑	1.330669096	✓	0.00414532
132	ENSGALG00000025996	MKI67	↑	1.327310352	✓	0.001506704
133	ENSGALG00000008713	BIRC5	↑	1.319178271	✓	0.004371918
134	ENSGALG00000028347	SDCBP2	↑	1.308996882	✓	0.005307204
135	ENSGALG00000003577	RNF19B	↑	1.286333279	✓	0.000899956
136	ENSGALG00000002909	CTGF	↑	1.280852029	✓	0.004613107
137	ENSGALG00000013033	CMBL	↑	1.277697825	✓	2.14081E-05
138	ENSGALG00000003085	CDK1	↑	1.263241511	✓	0.001294227
139	ENSGALG00000001255	ARHGEF37	↑	1.243027913	✓	0.001273956
140	ENSGALG00000003527	ARRDC2	↑	1.200030223	✓	0.00040186
141	ENSGALG00000005553	NLGN3	↑	1.199789071	✓	0.000208889
142	ENSGALG00000013811	NCAPH2	↑	1.18523842	✓	0.008697397
143	ENSGALG00000026152	GBP	↑	1.183913841	✓	0.000294843
144	ENSGALG00000015468		↑	1.13074611	✓	0.000590315
145	ENSGALG00000028939	AOC1	↑	1.130458922	✓	0.002087436
146	ENSGALG00000005408	BCMO1	↑	1.124302702	✓	0.005546813
147	ENSGALG00000007579	APBB1IP	↑	1.120194581	✓	0.009717239
148	ENSGALG00000023742		↑	1.103095294	✓	0.006108384
149	ENSGALG00000011747		↑	1.096722942	✓	2.85315E-07
150	ENSGALG00000008046	COQ10B	↑	1.093981426	✓	0.006275806
151	ENSGALG00000006226	KLHL3	↑	1.084758284	✓	1.14895E-05
152	ENSGALG00000025721	.	↑	1.075791368	✓	0.000228123
153	ENSGALG00000028664	CKS1B	↑	1.070111748	✓	0.003127764
154	ENSGALG00000023689	ASS1	↑	1.062140181	✓	0.003072612
155	ENSGALG00000006267	TPX2	↑	1.048852426	✓	0.005607343
156	ENSGALG00000027571	HIST1H2B7L2	↑	1.044799773	✓	0.007190948
157	ENSGALG00000021275	LTC4S	↑	1.038498077	✓	0.005031542
158	ENSGALG00000026165	TTC31	↑	1.027343662	✓	0.003365915
159	ENSGALG00000001398	.	↑	1.009177216	✓	0.001932108
160	ENSGALG00000011335	NHEJ1	↑	1.003948291	✓	0.003259151
161	ENSGALG00000025974	STAT4	↑	0.997845205	✓	0.00083862
162	ENSGALG00000014950		↑	0.996747907	✓	0.002131105
163	ENSGALG00000005099	ORM1	↑	0.996486062	✓	0.000369789
164	ENSGALG00000005541	GJB1	↑	0.976986504	✓	2.46091E-05
165	ENSGALG00000027106	.	↑	0.958241208	✓	0.003117511
166	ENSGALG00000005688	DNASE1L3	↑	0.951524803	✓	0.00415989
167	ENSGALG00000026510	.	↑	0.945920653	✓	0.00377097

168	ENSGALG00000021616	MARCKSL1	↑	0.945455088	✓	0.004602722
169	ENSGALG00000006471	SLCO2A1	↑	0.941463119	✓	0.009837302
170	ENSGALG00000002540	RGS2	↑	0.933426282	✓	0.000723771
171	ENSGALG00000003899	RAB17	↑	0.910279901	✓	0.000358128
172	ENSGALG00000027495	CARMIL3	↑	0.906282083	✓	0.002447856
173	ENSGALG00000012200	GCH1	↑	0.893287332	✓	0.002207536
174	ENSGALG00000006448	IRF9	↑	0.869407017	✓	0.001775108
175	ENSGALG00000028529	PTGDS	↑	0.826947834	✓	0.000959573
176	ENSGALG00000004521	GPX3	↑	0.81762586	✓	0.004705432
177	ENSGALG00000016289	DST	↑	0.813917976	✓	0.001041011
178	ENSGALG00000016224	MAOA	↑	0.796962066	✓	0.003712834
179	ENSGALG00000025842	TPM2	↑	0.787281167	✓	0.009207519
180	ENSGALG00000024011	LCN8	↑	0.787203036	✓	0.001628749
181	ENSGALG00000026269	TAP1	↑	0.770389091	✓	0.002567089
182	ENSGALG00000020688	CYP4B1L	↑	0.745910943	✓	0.006526243
183	ENSGALG00000002832	IFI35	↑	0.74583816	✓	0.006034872
184	ENSGALG00000000181		↑	0.731261787	✓	0.002311727
185	ENSGALG00000026646	C4	↑	0.720305656	✓	0.003482501
186	ENSGALG00000012285	BAIAP2L2	↑	0.718067779	✓	0.006989609
187	ENSGALG00000006283	THAP4	↑	0.706901369	✓	0.006754698
188	ENSGALG00000022586	HPX	↑	0.692610759	✓	0.005837961
189	ENSGALG00000027402	PRR15L	↑	0.681403662	✓	0.003305444
190	ENSGALG00000005795	CYP2H1	↑	0.66219686	✓	0.004195112
191	ENSGALG00000023608	.	↑	0.657563649	✓	0.006172929
192	ENSGALG00000013303	SLC27A1	↑	0.653626388	✓	0.00443613
193	ENSGALG00000028871	SLC38A3	↑	0.630430251	✓	0.002477298
194	ENSGALG00000010769	HPGD	↑	0.614648014	✓	0.004034877
195	ENSGALG00000008022	TAPBP	↑	0.608581243	✓	0.00612755
196	ENSGALG00000008469	SPINT1	↑	0.58700158	✓	0.007428629
197	ENSGALG00000004228	TRAF3IP1	↓	-0.596554343	✓	0.009257471
198	ENSGALG00000001220	HERPUD1	↓	-0.669312893	✓	0.008883981
199	ENSGALG00000004612	MTHFR	↓	-0.687809725	✓	0.001871268
200	ENSGALG00000005125	LSM14B	↓	-0.71891408	✓	0.007637154
201	ENSGALG00000014190	PNPLA3	↓	-0.752112765	✓	0.001760285
202	ENSGALG00000008152	SPATS2L	↓	-0.758785311	✓	0.007562393
203	ENSGALG00000023576	PIM3	↓	-0.769382877	✓	0.001858762
204	ENSGALG00000013814	IMPA2	↓	-0.775562962	✓	0.003405974
205	ENSGALG00000008780	CTBS	↓	-0.780407964	✓	0.007653276
206	ENSGALG00000003515	PECAM1	↓	-0.784603979	✓	0.009913052
207	ENSGALG00000015342	CEP97	↓	-0.811869716	✓	0.008687416
208	ENSGALG00000016828	GRTP1	↓	-0.821775345	✓	0.009686651
209	ENSGALG00000015998	PTDSS1	↓	-0.829818496	✓	0.008229714
210	ENSGALG00000016313	GCLC	↓	-0.832963407	✓	0.003642823
211	ENSGALG00000001443	PTPRU	↓	-0.869615265	✓	0.001843645
212	ENSGALG00000000227	DPYSL2	↓	-0.891094529	✓	0.003377546
213	ENSGALG00000027839	.	↓	-0.9056844	✓	0.009248124
214	ENSGALG00000008860	LONRF3	↓	-0.912153791	✓	0.003559901
215	ENSGALG00000025884	NABP1	↓	-0.913578588	✓	0.009678294
216	ENSGALG00000015792	ADAMTS1	↓	-0.96828589	✓	0.005054551
217	ENSGALG00000023435	GATM	↓	-0.970822673	✓	0.009800894
218	ENSGALG00000001603	NARF	↓	-1.001021533	✓	0.008032603
219	ENSGALG00000026250	DOK2	↓	-1.013086365	✓	0.005367396
220	ENSGALG00000026137	CDKN2B	↓	-1.017451129	✓	0.001269176
221	ENSGALG00000016345	KLHL15	↓	-1.044996732	✓	0.005845998
222	ENSGALG00000021344	.	↓	-1.078866007	✓	0.005985484
223	ENSGALG00000004033	GALNT10	↓	-1.114618927	✓	0.008001263
224	ENSGALG00000011285	NUDT4	↓	-1.129676593	✓	0.005831313

225	ENSGALG00000010522	ALDH18A1	↓	-1.167757092 ✓	0.007208007
226	ENSGALG00000013480	MTHFD2	↓	-1.190204004 ✓	0.00229656
227	ENSGALG00000011376	ANKRD9	↓	-1.213065618 ✓	0.008368805
228	ENSGALG00000004813	IARS	↓	-1.236055493 ✓	0.002088888
229	ENSGALG00000006521	TRPM5	↓	-1.246603882 ✓	0.006986694
230	ENSGALG00000009409	MYNN	↓	-1.262075547 ✓	0.009091016
231	ENSGALG00000004365	WDR66	↓	-1.304369839 ✓	0.001042404
232	ENSGALG00000012223	ZRANB3	↓	-1.310676905 ✓	0.005486975
233	ENSGALG00000016210	ST3GAL1	↓	-1.31098556 ✓	0.00608187
234	ENSGALG00000008795	GPAM	↓	-1.326425589 ✓	0.005572268
235	ENSGALG00000008361	THBD	↓	-1.339640336 ✓	0.009576954
236	ENSGALG00000009748	ASNS	↓	-1.348703323 ✓	0.006384616
237	ENSGALG00000013624	FAM65B	↓	-1.42676445 ✓	0.000996892
238	ENSGALG00000009020	JAG1	↓	-1.558685298 ✓	1.46358E-05
239	ENSGALG00000003466	AKR1B1L	↓	-1.609341933 ✓	0.005518215
240	ENSGALG00000015820	CA13	↓	-1.634213815 ✓	0.003273899
241	ENSGALG00000009725	SCG5	↓	-1.708084092 ✓	0.000268256
242	ENSGALG00000003456	CA12	↓	-1.715514304 ✓	0.00312268
243	ENSGALG00000015556	AFAP1	↓	-1.734967008 ✓	0.005477097
244	ENSGALG00000017197	CNTN5	↓	-1.753222056 ✓	0.006448293
245	ENSGALG00000011446	TNFAIP2	↓	-1.785607097 ✓	0.000751431
246	ENSGALG00000002923	.	↓	-1.794342762 ✓	0.007815672
247	ENSGALG00000010391	MMRN1	↓	-1.839975257 ✓	0.00066759
248	ENSGALG00000016262	XK	↓	-1.892906666 ✓	0.009939857
249	ENSGALG00000002437	FAM69B	↓	-1.901671995 ✓	0.00342469
250	ENSGALG00000014907	DCBLD1	↓	-1.928134624 ✓	0.000155627
251	ENSGALG00000017326	ARHGEF17	↓	-1.954450116 ✓	0.005708336
252	ENSGALG00000027054	.	↓	-1.955776521 ✓	0.006099836
253	ENSGALG00000023933	G0S2	↓	-2.031395466 ✓	0.003165331
254	ENSGALG00000012547	ACVR1C	↓	-2.057012027 ✓	0.003964789
255	ENSGALG00000015057	DDO	↓	-2.115701746 ✓	0.001830483
256	ENSGALG00000009042	ISM1	↓	-2.168356834 ✓	0.002543493
257	ENSGALG00000028250	ANKRD22	↓	-2.232208276 ✓	7.0467E-05
258	ENSGALG00000014277	RHOH	↓	-2.288910199 ✓	0.005002353
259	ENSGALG00000006997	BRINP1	↓	-2.320079318 ✓	3.72603E-09
260	ENSGALG00000012660	KIAA0319	↓	-2.431868636 ✓	0.008087642
261	ENSGALG00000004704	.	↓	-2.433800688 ✓	0.0017954
262	ENSGALG00000017836	.	↓	-2.551201049 ✓	0.00967938
263	ENSGALG00000006112	SCN5A	↓	-2.568771126 ✓	0.008445907
264	ENSGALG00000012494	A5HUM9_CHICK	↓	-2.844792318 ✓	0.001991093
265	ENSGALG00000016296	GFRAL	↓	-2.869000889 ✓	0.000161436
266	ENSGALG00000015734	ZC2HC1A	↓	-2.925723111 ✓	5.51656E-05
267	ENSGALG00000011202	COLQ	↓	-2.956548145 ✓	0.002023076
268	ENSGALG00000007948	LRRC4C	↓	-3.12709993 ✓	0.001716887
269	ENSGALG00000011598	NEK11	↓	-3.21191134 ✓	0.004142698
270	ENSGALG00000007426	MKX	↓	-3.294941211 ✓	0.001045803
271	ENSGALG00000012312	GCAT	↓	-3.482426232 ✓	0.005695969
272	ENSGALG00000009758	SLC7A11	↓	-3.565272239 ✓	0.006784804
273	ENSGALG00000016279	RAB23	↓	-3.58038778 ✓	0.002755109
274	ENSGALG00000026535	.	↓	-3.704717087 ✓	0.008958571
275	ENSGALG00000025465	MIR1458	↓	-3.86538854 ✓	0.007169369
276	ENSGALG00000010232	VRTN	↓	-3.873076234 ✓	0.007183202
277	ENSGALG00000028071	.	↓	-3.873338084 ✓	0.005619303
278	ENSGALG00000017879	gga-mir-3535	↓	-3.899715093 ✓	0.008733327
279	ENSGALG00000027389	RGS7BP	↓	-3.911093675 ✓	0.002857479
280	ENSGALG00000025268	MIR1658	↓	-3.952996868 ✓	0.008874384
281	ENSGALG00000016571	GLRA2	↓	-4.022947678 ✓	0.005127191

282	ENSGALG00000015276	.	-4.141259412	0.007931817
283	ENSGALG00000004128	CYSLTR1	-4.215621078	0.004020725
284	ENSGALG00000025564	.	-4.2376472	0.007799682
285	ENSGALG00000013183	TERT	-4.346597894	0.004386673
286	ENSGALG00000005762	.	-4.354632297	0.006354043
287	ENSGALG00000018276	MIR126	-4.611976704	0.002454974
288	ENSGALG00000020492	MUC6	-4.706841564	0.001103898
289	ENSGALG00000011808	CCR9	-4.966768981	0.000437094
290	ENSGALG00000007468	HBAA	-5.167195331	0.001282141

Table S3. The KEGG pathways

KeggID	KeggPathway	Number of Genes
path:gga01100	Metabolic pathways	24
path:gga05168	Herpes simplex infection	6
path:gga05164	Influenza A	5
path:gga04110	Cell cycle	5
path:gga04371	Apelin signaling pathway	4
path:gga04120	Ubiquitin mediated proteolysis	4
path:gga04080	Neuroactive ligand-receptor interaction	4
path:gga04514	Cell adhesion molecules (CAMs)	4
path:gga04530	Tight junction	4
path:gga04218	Cellular senescence	4
path:gga04068	FoxO signaling pathway	4
path:gga00250	Alanine, aspartate and glutamate metabolism	3
path:gga00590	Arachidonic acid metabolism	3
path:gga04261	Adrenergic signaling in cardiomyocytes	3
path:gga04910	Insulin signaling pathway	3
path:gga00564	Glycerophospholipid metabolism	3
path:gga00330	Arginine and proline metabolism	3
path:gga00910	Nitrogen metabolism	3
path:gga03320	PPAR signaling pathway	3
path:gga00670	One carbon pool by folate	2
path:gga04115	p53 signaling pathway	2
path:gga04216	Ferroptosis	2
path:gga00790	Folate biosynthesis	2
path:gga04217	Necroptosis	2
path:gga02010	ABC transporters	2
path:gga04145	Phagosome	2
path:gga00512	Mucin type O-glycan biosynthesis	2
path:gga03460	Fanconi anemia pathway	2
path:gga00480	Glutathione metabolism	2
path:gga04114	Oocyte meiosis	2
path:gga00561	Glycerolipid metabolism	2
path:gga00260	Glycine, serine and threonine metabolism	2
path:gga00230	Purine metabolism	2
path:gga00830	Retinol metabolism	2
path:gga04144	Endocytosis	2
path:gga01230	Biosynthesis of amino acids	2
path:gga04260	Cardiac muscle contraction	2
path:gga04621	NOD-like receptor signaling pathway	2
path:gga04920	Adipocytokine signaling pathway	2
path:gga00240	Pyrimidine metabolism	2
path:gga04350	TGF-beta signaling pathway	2
path:gga04914	Progesterone-mediated oocyte maturation	2
path:gga04141	Protein processing in endoplasmic reticulum	1
path:gga01200	Carbon metabolism	1
path:gga00140	Steroid hormone biosynthesis	1
path:gga04933	AGE-RAGE signaling pathway in diabetic complications	1
path:gga04620	Toll-like receptor signaling pathway	1
path:gga00600	Sphingolipid metabolism	1
path:gga04060	Cytokine-cytokine receptor interaction	1
path:gga04672	Intestinal immune network for IgA production	1
path:gga00533	Glycosaminoglycan biosynthesis - keratan sulfate	1
path:gga00603	Glycosphingolipid biosynthesis - globo and isoglobo series	1
path:gga00604	Glycosphingolipid biosynthesis - ganglio series	1

path:gga00270	Cysteine and methionine metabolism	1
path:gga00040	Pentose and glucuronate interconversions	1
path:gga00051	Fructose and mannose metabolism	1
path:gga00052	Galactose metabolism	1
path:gga00860	Porphyrin and chlorophyll metabolism	1
path:gga00340	Histidine metabolism	1
path:gga00350	Tyrosine metabolism	1
path:gga00360	Phenylalanine metabolism	1
path:gga00380	Tryptophan metabolism	1
path:gga00982	Drug metabolism - cytochrome P450	1
path:gga00562	Inositol phosphate metabolism	1
path:gga04070	Phosphatidylinositol signaling system	1
path:gga04330	Notch signaling pathway	1
path:gga00760	Nicotinate and nicotinamide metabolism	1
path:gga00220	Arginine biosynthesis	1
path:gga04146	Peroxisome	1
path:gga00970	Aminoacyl-tRNA biosynthesis	1
path:gga03030	DNA replication	1
path:gga04510	Focal adhesion	1
path:gga04810	Regulation of actin cytoskeleton	1
path:gga00010	Glycolysis / Gluconeogenesis	1
path:gga00020	Citrate cycle (TCA cycle)	1
path:gga00620	Pyruvate metabolism	1
path:gga04020	Calcium signaling pathway	1
path:gga04210	Apoptosis	1
path:gga04622	RIG-I-like receptor signaling pathway	1
path:gga04540	Gap junction	1

Table S4. The edge details in protein-protein interaction network

shared name	coexpression	cooccurrence	databases	experiments	fusion	neighborhood	score	textmining
SGPP2 (pp) CERKL	0.086			0.163		0.07	0.713	0.645
MX1 (pp) TLR3	0.736			0.096			0.932	0.739
MX1 (pp) EPSTI1	0.828						0.858	0.209
MX1 (pp) RSAD2	0.774						0.959	0.827
DDO (pp) PCK1	0.058		0.8			0.069	0.84	0.196
USP18 (pp) RSAD2	0.817						0.925	0.606
USP18 (pp) MX1	0.835			0.122			0.948	0.67
USP18 (pp) OASL	0.825			0.38			0.954	0.616
TTK (pp) CKS1B	0.781			0.562			0.918	0.214
TTK (pp) MKI67	0.903						0.908	0.084
TTK (pp) CDK1	0.997			0.367			0.998	0.253
TTK (pp) UBE2C	0.835						0.846	0.103
TTK (pp) TPX2	0.894						0.911	0.193
TTK (pp) NUF2	0.895			0.369			0.976	0.665
TTK (pp) BIRC5	0.847						0.868	0.176
TTK (pp) CKAP2	0.958						0.958	
TTK (pp) BORA	0.726						0.727	0.046
TTK (pp) PBK	0.903						0.938	0.392
STOML1 (pp) RNF17				0.16			0.828	0.803
BIRC5 (pp) CKS1B	0.935						0.941	0.134
BIRC5 (pp) MKI67	0.777						0.849	0.35
BIRC5 (pp) CDK1	0.959		0.9	0.16			0.997	0.341
BIRC5 (pp) UBE2C	0.891			0.252			0.943	0.366
BIRC5 (pp) TPX2	0.906						0.931	0.292
BIRC5 (pp) APITD1	0.328		0.9				0.93	
BIRC5 (pp) NUF2	0.859						0.888	0.236
PECAM1 (pp) MKI67							0.792	0.792
ASPM (pp) CKS1B	0.748						0.754	0.065
ASPM (pp) MKI67	0.959						0.969	0.281
ASPM (pp) CDK1	0.996			0.042			0.997	0.481
ASPM (pp) UBE2C	0.844						0.858	0.132
ASPM (pp) CCNB3	0.805						0.823	0.13
ASPM (pp) TPX2	0.981						0.982	0.056
ASPM (pp) NUF2	0.744						0.773	0.15
ASPM (pp) BIRC5	0.729						0.763	0.162
ASPM (pp) CKAP2	0.989						0.993	0.399
ASPM (pp) PBK	0.891						0.907	0.19
ASPM (pp) TTK	0.996						0.996	0.192
ASPM (pp) NDC80	0.99						0.99	0.055
ASPM (pp) CDCA3	0.962						0.965	0.121
ASPM (pp) NCAPG	0.995						0.996	0.345
ASPM (pp) FOXM1	0.787						0.79	0.055
ASPM (pp) CENPE	0.996			0.042			0.997	0.389
ASPM (pp) KIF15	0.918			0.042			0.969	0.636
ASPM (pp) DEPDC1	0.886						0.915	0.29
ASPM (pp) KIF23	0.981			0.042			0.99	0.512
ASPM (pp) CEP55	0.986						0.988	0.212
ASPM (pp) RACGAP1	0.727						0.76	0.159
ASPM (pp) KNTC1	0.738			0.237			0.814	0.147
ASPM (pp) KIF4A	0.884			0.042			0.947	0.56
ASPM (pp) KIF20A	0.926			0.042			0.954	0.41
TAPBP (pp) GBP	0.784						0.784	
TAPBP (pp) TAP1	0.123		0.9				0.908	

CKAP2 (pp) MKI67	0.9			0.903	0.066
CKAP2 (pp) CDK1	0.93			0.933	0.086
CKAP2 (pp) UBE2C	0.829		0.161	0.85	0.042
CKAP2 (pp) TPX2	0.867			0.87	0.068
CKAP2 (pp) NUF2	0.828			0.837	0.09
CKAP2 (pp) BIRC5	0.781			0.781	
OASL (pp) TLR3	0.362		0.087	0.704	0.533
OASL (pp) UBE2L6	0.057		0.62	0.737	0.327
OASL (pp) GBP	0.723			0.781	0.241
OASL (pp) EPSTI1	0.793			0.851	0.311
OASL (pp) RSAD2	0.697			0.922	0.754
OASL (pp) MX1	0.999		0.122	0.999	0.824
FOXM1 (pp) MKI67	0.893		0.059	0.93	0.356
FOXM1 (pp) CDK1	0.785	0.9	0.252	0.994	0.679
FOXM1 (pp) UBE2C	0.684		0.097	0.859	0.546
FOXM1 (pp) TPX2	0.792			0.815	0.148
FOXM1 (pp) BIRC5	0.669			0.791	0.393
FOXM1 (pp) PBK	0.695		0.112	0.744	0.128
FOXM1 (pp) TTK	0.76			0.778	0.112
FOXM1 (pp) NDC80	0.839		0.051	0.856	0.134
FOXM1 (pp) CDCA3	0.779			0.831	0.267
FOXM1 (pp) NCAPG	0.813			0.818	0.065
IARS (pp) ASNS	0.222		0.662	0.766	0.184
CDK1 (pp) CKS1B	0.998		0.87	0.999	0.6
CDK1 (pp) MKI67	0.819		0.161	0.94	0.637
KIF4A (pp) CKS1B	0.527			0.768	0.53
KIF4A (pp) MKI67	0.767		0.055	0.825	0.269
KIF4A (pp) CDK1	0.935		0.159	0.971	0.509
KIF4A (pp) UBE2C	0.864		0.087	0.897	0.237
KIF4A (pp) TPX2	0.953			0.963	0.256
KIF4A (pp) NUF2	0.83		0.16	0.868	0.155
KIF4A (pp) BIRC5	0.865		0.047	0.916	0.399
KIF4A (pp) SKA3	0.765			0.767	0.049
KIF4A (pp) CKAP2	0.806			0.861	0.313
KIF4A (pp) PBK	0.851		0.112	0.877	0.141
KIF4A (pp) TTK	0.776		0.068	0.806	0.143
KIF4A (pp) NDC80	0.895		0.164	0.931	0.279
KIF4A (pp) CDCA3	0.99			0.991	0.133
KIF4A (pp) NCAPG	0.695		0.052	0.759	0.234
KIF4A (pp) CENPE	0.89	0.9		0.991	0.275
KIF4A (pp) KIF15	0.561	0.9		0.967	0.308
KIF4A (pp) KIF2C	0.825	0.9		0.985	0.234
KIF4A (pp) KIF23	0.916	0.9	0.112	0.994	0.393
KIF4A (pp) INCENP	0.903		0.068	0.977	0.769
KIF4A (pp) CEP55	0.568		0.084	0.724	0.359
KIF4A (pp) RACGAP1	0.807	0.9	0.227	0.991	0.499
KIF4A (pp) GTSE1	0.618			0.725	0.311
UBE2T (pp) CDK1	0.757		0.159	0.805	0.121
UBE2T (pp) APITD1	0.544	0.9		0.963	0.264
UBE2T (pp) BIRC5	0.646		0.369	0.783	0.106
UBE2T (pp) PTTG1	0.753			0.76	0.068
KIF18B (pp) MKI67	0.844		0.055	0.846	
KIF18B (pp) UBE2C	0.725		0.087	0.765	0.14
KIF18B (pp) NDC80	0.452		0.369	0.772	0.393
KIF18B (pp) FOXM1	0.728		0.062	0.733	

KIF18B (pp) CENPE	0.384	0.9	0.249			0.959	0.236
KIF18B (pp) KIF15	0.635	0.9				0.968	0.21
KIF18B (pp) KIF2C	0.282	0.9	0.258			0.953	0.235
KIF18B (pp) KIF23	0.421	0.9				0.949	0.194
KIF18B (pp) RACGAP1	0.305	0.9	0.227			0.955	0.271
KIF18B (pp) KIF4A	0.62	0.9				0.966	0.189
KIF18B (pp) KIF20A	0.738	0.9				0.974	0.085
KIF18B (pp) ASPM	0.72		0.042			0.751	0.149
NDC80 (pp) CKS1B	0.697					0.798	0.362
NDC80 (pp) MKI67	0.879					0.882	0.067
NDC80 (pp) CDK1	0.996	0.9				0.999	0.552
NDC80 (pp) UBE2C	0.845		0.052			0.868	0.172
NDC80 (pp) CCNB3	0.639		0.096			0.703	0.162
NDC80 (pp) TPX2	0.92					0.936	0.232
NDC80 (pp) APITD1	0.178	0.9				0.973	0.708
NDC80 (pp) NUF2	0.878	0.72	0.963			0.999	0.962
NDC80 (pp) BIRC5	0.838	0.9				0.986	0.251
NDC80 (pp) SKA3	0.795					0.947	0.751
NDC80 (pp) CKAP2	0.93					0.93	0.043
NDC80 (pp) PBK	0.933					0.935	0.075
NDC80 (pp) TTK	0.995		0.59			0.999	0.635
ASNS (pp) ASS1	0.629	0.9	0.16		0.045	0.988	0.671
UBE2C (pp) CKS1B	0.908		0.042			0.932	0.299
UBE2C (pp) MKI67	0.796					0.856	0.321
UBE2C (pp) CDK1	0.976	0.9	0.136			0.998	0.521
ARHGEF37 (pp) RHOB	0.057	0.9	0.308			0.933	0.098
ARHGEF37 (pp) RHOH		0.9	0.308			0.932	0.098
LOC431660 (pp) PCK1	0.785					0.785	
SMYD1 (pp) MYL2	0.723		0.066			0.794	0.269
SOCS1 (pp) STAT4			0.153			0.844	0.823
CCNB3 (pp) CKS1B	0.547		0.693			0.9	0.341
CCNB3 (pp) CDK1	0.989	0.8	0.791	0.002		0.999	0.74
CCNB3 (pp) UBE2C	0.663		0.16			0.8	0.351
BORA (pp) CDK1	0.38	0.9				0.976	0.644
IGFBP1 (pp) C4	0.058	0.9				0.904	0.07
KNTC1 (pp) CDK1	0.776	0.9				0.977	0.071
KNTC1 (pp) APITD1	0.145	0.9				0.923	0.172
KNTC1 (pp) NUF2	0.521					0.722	0.444
KNTC1 (pp) BIRC5	0.586	0.9				0.96	0.132
KNTC1 (pp) TTK	0.713					0.741	0.134
KNTC1 (pp) NDC80	0.654	0.9				0.985	0.602
KNTC1 (pp) CENPE	0.713	0.9				0.99	0.693
KNTC1 (pp) KIF15	0.692					0.701	0.068
KNTC1 (pp) KIF2C	0.381	0.9				0.941	0.124
KNTC1 (pp) KIF23	0.668					0.701	0.138
KNTC1 (pp) INCENP	0.488	0.9				0.962	0.334
KNTC1 (pp) CENPI	0.379	0.9				0.959	0.394
KNTC1 (pp) C15ORF23	0.505					0.718	0.453
PTTG1 (pp) CDK1	0.926		0.161			0.985	0.783
PTTG1 (pp) UBE2C	0.93	0.9				0.996	0.574
PTTG1 (pp) TPX2	0.881					0.894	0.145
PTTG1 (pp) NUF2	0.746					0.83	0.359
PTTG1 (pp) BIRC5	0.899					0.917	0.215
PTTG1 (pp) PBK	0.879					0.887	0.102
PTTG1 (pp) TTK	0.759					0.803	0.214

PTTG1 (pp) NDC80	0.786			0.848	0.317
PTTG1 (pp) CDCA3	0.925			0.933	0.133
PTTG1 (pp) NCAPG	0.757			0.804	0.225
PTTG1 (pp) FOXM1	0.419			0.748	0.583
PTTG1 (pp) CENPE	0.476			0.723	0.493
PTTG1 (pp) CEP55	0.657			0.717	0.208
PTTG1 (pp) KIF4A	0.697			0.91	0.715
PTTG1 (pp) KIF20A	0.873			0.885	0.135
PTTG1 (pp) ASPM	0.711			0.796	0.324
CYSLTR1 (pp) LTC4S				0.823	0.823
RNF19B (pp) UBE2C	0.055	0.9	0.238	0.923	0.057
RNF19B (pp) USP18	0.055			0.729	0.725
RNF19B (pp) RNF213	0.057	0.9		0.968	0.689
RNF19B (pp) KLHL3	0.057	0.9		0.901	
CENPI (pp) CDK1	0.425	0.9		0.95	0.206
CENPI (pp) HIST1H2B7		0.9		0.9	0.046
CENPI (pp) APITD1	0.204	0.9		0.988	0.863
CENPI (pp) NUF2	0.481			0.904	0.823
CENPI (pp) BIRC5	0.428	0.9		0.94	0.046
CENPI (pp) NDC80	0.596	0.9	0.167	0.993	0.834
CENPI (pp) NCAPG	0.658			0.787	0.403
CENPI (pp) CENPE	0.409	0.9		0.98	0.694
CENPI (pp) KIF2C	0.365	0.9		0.961	0.442
CENPI (pp) INCENP	0.396	0.9		0.958	0.372
CENPI (pp) RACGAP1	0.436			0.722	0.528
APITD1 (pp) CDK1	0.339	0.9		0.945	0.241
APITD1 (pp) HIST1H2B7		0.9		0.9	0.045
CENPE (pp) MKI67	0.91		0.055	0.936	0.31
CENPE (pp) CDK1	0.962	0.9	0.161	0.998	0.574
CENPE (pp) UBE2C	0.786		0.087	0.874	0.408
CENPE (pp) TPX2	0.882			0.943	0.537
CENPE (pp) APITD1	0.092	0.9		0.972	0.717
CENPE (pp) NUF2	0.805		0.247	0.97	0.812
CENPE (pp) BIRC5	0.718	0.9	0.047	0.975	0.205
CENPE (pp) SKA3	0.738			0.908	0.662
CENPE (pp) CKAP2	0.937		0.042	0.951	0.262
CENPE (pp) PBK	0.839		0.112	0.863	0.119
CENPE (pp) TTK	0.997		0.068	0.998	0.512
CENPE (pp) NDC80	0.937	0.9	0.369	0.999	0.884
CENPE (pp) CDCA3	0.902			0.91	0.128
CENPE (pp) NCAPG	0.992		0.052	0.994	0.255
CENPE (pp) FOXM1	0.723		0.062	0.752	0.122
KLHL3 (pp) UBE2C	0.058	0.9	0.111	0.908	
KLHL3 (pp) RNF213	0.056	0.9		0.901	
ISG12-2 (pp) TLR3	0.738			0.848	0.444
ISG12-2 (pp) GBP	0.783			0.803	0.13
ISG12-2 (pp) EPSTI1	0.775			0.875	0.471
ISG12-2 (pp) RSAD2	0.361			0.786	0.68
ISG12-2 (pp) MX1	0.884			0.959	0.666
ISG12-2 (pp) OASL	0.999			0.999	0.815
KIF2C (pp) CDK1	0.8	0.9	0.159	0.989	0.444
KIF2C (pp) UBE2C	0.824		0.087	0.863	0.216
KIF2C (pp) TPX2	0.826			0.868	0.271
KIF2C (pp) APITD1	0.099	0.9		0.96	0.601
KIF2C (pp) NUF2	0.688		0.16	0.823	0.38

KIF2C (pp) BIRC5	0.615		0.9	0.047	0.962	0.097
KIF2C (pp) SKA3	0.528				0.744	0.479
KIF2C (pp) CKAP2	0.788				0.788	
KIF2C (pp) PBK	0.791			0.112	0.809	0.052
KIF2C (pp) TTK	0.728			0.068	0.776	0.187
KIF2C (pp) NDC80	0.671		0.9	0.164	0.992	0.758
KIF2C (pp) CDCA3	0.827				0.831	0.065
KIF2C (pp) CENPE	0.819		0.9	0.145	0.988	0.321
KIF2C (pp) KIF15	0.44		0.9		0.953	0.234
DEPDC1 (pp) MKI67	0.755				0.755	
DEPDC1 (pp) CDK1	0.792				0.795	0.056
DEPDC1 (pp) CKAP2	0.811				0.819	0.084
DEPDC1 (pp) PBK	0.72				0.774	0.227
DEPDC1 (pp) TTK	0.819				0.851	0.21
DEPDC1 (pp) NDC80	0.836				0.838	0.053
DEPDC1 (pp) CDCA3	0.369				0.792	0.684
DEPDC1 (pp) NCAPG	0.704				0.713	0.068
DEPDC1 (pp) CENPE	0.799				0.84	0.236
DEPDC1 (pp) KIF15	0.76				0.816	0.268
INCENP (pp) CDK1	0.864		0.9	0.122	0.998	0.87
INCENP (pp) TPX2	0.774				0.918	0.651
INCENP (pp) APITD1	0.233		0.9		0.973	0.681
INCENP (pp) NUF2	0.543				0.779	0.536
INCENP (pp) BIRC5	0.54		0.9	0.355	0.989	0.699
INCENP (pp) CKAP2	0.758				0.813	0.26
INCENP (pp) PBK	0.753				0.791	0.189
INCENP (pp) TTK	0.68				0.773	0.322
INCENP (pp) NDC80	0.724		0.9		0.995	0.844
INCENP (pp) CENPE	0.826		0.9	0.068	0.996	0.826
INCENP (pp) KIF15	0.194			0.068	0.74	0.682
INCENP (pp) KIF2C	0.708		0.9	0.068	0.993	0.785
INCENP (pp) KIF23	0.842			0.143	0.963	0.749
RHOB (pp) SPEG				0.677	0.715	0.155
RHOB (pp) ARHGEF17	0.085		0.9	0.112	0.924	0.175
KIF23 (pp) CDK1	0.992			0.159	0.998	0.787
KIF23 (pp) UBE2C	0.733			0.087	0.781	0.173
KIF23 (pp) CCNB3	0.84			0.132	0.883	0.226
KIF23 (pp) TPX2	0.853				0.913	0.432
KIF23 (pp) NUF2	0.768			0.16	0.819	0.147
KIF23 (pp) BIRC5	0.62			0.066	0.745	0.341
KIF23 (pp) SKA3	0.737				0.737	
KIF23 (pp) CKAP2	0.667				0.777	0.358
KIF23 (pp) PBK	0.857			0.112	0.874	0.092
KIF23 (pp) TTK	0.852			0.068	0.872	0.151
KIF23 (pp) NDC80	0.923			0.164	0.941	0.161
KIF23 (pp) CDCA3	0.841				0.86	0.152
KIF23 (pp) NCAPG	0.846			0.052	0.874	0.208
KIF23 (pp) FOXM1	0.808			0.043	0.832	0.159
KIF23 (pp) CENPE	0.836		0.9		0.988	0.33
KIF23 (pp) KIF15	0.723		0.9		0.98	0.336
KIF23 (pp) KIF2C	0.731		0.9		0.976	0.21
RHOH (pp) SPEG				0.677	0.715	0.155
RHOH (pp) ARHGEF17			0.9	0.112	0.91	0.073
RHOH (pp) RHOB		0.074	0.9		0.906	0.074
RHOH (pp) DEPDC1B	0.055		0.9		0.901	

WDR31 (pp) OASL	0.192			0.605		0.711	0.168
SBSPON (pp) ADAMTS1			0.9			0.9	
CEP55 (pp) MKI67	0.858					0.917	0.439
CEP55 (pp) CDK1	0.936			0.133		0.957	0.277
CEP55 (pp) UBE2C	0.804					0.873	0.381
CEP55 (pp) TPX2	0.781					0.805	0.148
CEP55 (pp) NUF2	0.762					0.839	0.349
CEP55 (pp) BIRC5	0.7					0.76	0.233
CEP55 (pp) CKAP2	0.79					0.884	0.47
CEP55 (pp) PBK	0.888					0.909	0.227
CEP55 (pp) TTK	0.948					0.959	0.235
CEP55 (pp) NDC80	0.851					0.859	0.093
CEP55 (pp) NCAPG	0.867					0.883	0.152
CEP55 (pp) FOXM1	0.719					0.858	0.515
CEP55 (pp) CENPE	0.802			0.084		0.879	0.388
CEP55 (pp) KIF15	0.852			0.084		0.902	0.337
CEP55 (pp) DEPDC1	0.825					0.889	0.393
CEP55 (pp) KIF23	0.79			0.268		0.963	0.778
CCR9 (pp) GGCL1			0.9	0.162		0.921	0.14
PBK (pp) MKI67	0.854			0.048		0.861	0.077
PBK (pp) CDK1	0.986	0.274		0.161		0.995	0.483
PBK (pp) UBE2C	0.901			0.161		0.917	0.084
PBK (pp) TPX2	0.923			0.068		0.933	0.143
PBK (pp) NUF2	0.913					0.925	0.179
PBK (pp) BIRC5	0.903			0.238		0.935	0.186
PBK (pp) SKA3	0.768					0.768	
PBK (pp) CKAP2	0.844					0.848	0.066
NUF2 (pp) CKS1B	0.785					0.795	0.084
NUF2 (pp) MKI67	0.733					0.761	0.144
NUF2 (pp) CDK1	0.957					0.969	0.303
NUF2 (pp) UBE2C	0.773					0.816	0.225
NUF2 (pp) TPX2	0.891					0.932	0.406
IMPA2 (pp) ANKRD22						0.79	0.79
KIF15 (pp) MKI67	0.855			0.161		0.906	0.291
KIF15 (pp) CDK1	0.747			0.159		0.917	0.64
KIF15 (pp) UBE2C	0.71			0.087		0.726	0.05
KIF15 (pp) TPX2	0.748					0.944	0.79
KIF15 (pp) NUF2	0.629			0.16		0.816	0.458
KIF15 (pp) BIRC5	0.677			0.047		0.724	0.175
KIF15 (pp) CKAP2	0.812					0.814	0.052
KIF15 (pp) PBK	0.753			0.112		0.794	0.136
KIF15 (pp) TTK	0.906			0.068		0.925	0.208
KIF15 (pp) NDC80	0.896			0.164		0.944	0.411
KIF15 (pp) CDCA3	0.684					0.716	0.137
KIF15 (pp) NCAPG	0.993			0.052		0.995	0.379
KIF15 (pp) FOXM1	0.752			0.043		0.757	0.058
KIF15 (pp) CENPE	0.925		0.9			0.993	0.258
ORM1 (pp) AOC1			0.9			0.9	
ORM1 (pp) HPX	0.547					0.801	0.578
ORM1 (pp) MMRN1			0.9			0.903	0.074
ALDH18A1 (pp) CKS1B	0.064			0.869		0.872	
ALDH18A1 (pp) ASS1	0.095				0.439	0.912	0.84
ALDH18A1 (pp) MTHFD2	0.206			0.043	0.428	0.829	0.653
TPX2 (pp) MKI67	0.849					0.864	0.132
TPX2 (pp) CDK1	0.921		0.9	0.042		0.997	0.698

TPX2 (pp) UBE2C	0.929		0.065		0.956	0.396
MTHFR (pp) MTHFD2	0.138	0.9		0.221	0.966	0.566
MTHFR (pp) GCH1	0.088			0.169	0.704	0.642
NCAPH2 (pp) HIST1H2B7		0.9			0.9	
NCAPH2 (pp) NCAPG	0.229		0.086		0.789	0.725
RACGAP1 (pp) CDK1	0.87		0.16		0.945	0.541
RACGAP1 (pp) TPX2	0.815				0.835	0.148
RACGAP1 (pp) BIRC5	0.684				0.758	0.266
RACGAP1 (pp) PBK	0.77		0.068		0.789	0.092
RACGAP1 (pp) RHOB		0.9	0.167		0.95	0.457
RACGAP1 (pp) TTK	0.673				0.725	0.193
RACGAP1 (pp) NDC80	0.759				0.805	0.224
RACGAP1 (pp) NCAPG	0.762				0.776	0.094
RACGAP1 (pp) RHOH		0.9	0.167		0.921	0.136
RACGAP1 (pp) CENPE	0.505	0.9	0.227		0.984	0.649
RACGAP1 (pp) KIF15	0.554	0.9	0.227		0.975	0.38
RACGAP1 (pp) KIF2C	0.441	0.9	0.227		0.965	0.289
RACGAP1 (pp) KIF23	0.895	0.9	0.856		0.999	0.838
RACGAP1 (pp) INCENP	0.658				0.878	0.657
RACGAP1 (pp) CEP55	0.74		0.266		0.955	0.783
RNF213 (pp) UBE2C		0.9			0.9	
HPX (pp) HBAA	0.054	0.9			0.916	0.189
SKA3 (pp) CDK1	0.795				0.867	0.38
SKA3 (pp) TPX2	0.819				0.822	0.055
SKA3 (pp) NUF2	0.684				0.878	0.63
NCAPG (pp) CKS1B	0.977				0.977	0.071
NCAPG (pp) MKI67	0.851				0.851	
NCAPG (pp) CDK1	0.998		0.143		0.998	0.162
NCAPG (pp) UBE2C	0.778				0.795	0.115
NCAPG (pp) TPX2	0.866				0.885	0.173
NCAPG (pp) NUF2	0.887				0.907	0.212
NCAPG (pp) BIRC5	0.786		0.257		0.846	0.111
NCAPG (pp) CKAP2	0.746				0.828	0.352
NCAPG (pp) PBK	0.883				0.897	0.158
NCAPG (pp) TTK	0.994				0.995	0.177
NCAPG (pp) NDC80	0.992				0.994	0.279
CDCA3 (pp) CKS1B	0.725				0.726	0.043
CDCA3 (pp) MKI67	0.697				0.793	0.346
CDCA3 (pp) CDK1	0.995				0.996	0.337
CDCA3 (pp) UBE2C	0.918				0.933	0.219
CDCA3 (pp) CCNB3	0.733				0.74	0.065
CDCA3 (pp) TPX2	0.875				0.886	0.129
CDCA3 (pp) NUF2	0.827				0.891	0.396
CDCA3 (pp) BIRC5	0.95				0.956	0.153
CDCA3 (pp) SKA3	0.753				0.753	
CDCA3 (pp) CKAP2	0.918				0.937	0.269
CDCA3 (pp) PBK	0.968				0.977	0.301
CDCA3 (pp) TTK	0.882				0.89	0.102
CDCA3 (pp) NDC80	0.914				0.914	
SLBP (pp) CDK1	0.765		0.16		0.842	0.263
DNA2 (pp) CDK1	0.45		0.077		0.781	0.604
DNA2 (pp) KIF4A	0.761				0.761	
KIF20A (pp) MKI67	0.865		0.055		0.898	0.262
KIF20A (pp) CDK1	0.989		0.159		0.997	0.706
KIF20A (pp) UBE2C	0.985		0.087		0.99	0.387

KIF20A (pp) TPX2	0.956			0.97	0.347
KIF20A (pp) NUF2	0.726		0.16	0.867	0.471
KIF20A (pp) SKA3	0.763			0.763	
KIF20A (pp) CKAP2	0.851			0.906	0.395
KIF20A (pp) PBK	0.949		0.112	0.958	0.145
KIF20A (pp) TTK	0.91		0.068	0.925	0.182
KIF20A (pp) NDC80	0.92		0.164	0.936	0.122
KIF20A (pp) CDCA3	0.893			0.941	0.47
KIF20A (pp) NCAPG	0.925		0.052	0.934	0.146
KIF20A (pp) FOXM1	0.768		0.043	0.88	0.506
KIF20A (pp) CENPE	0.951	0.9		0.996	0.252
KIF20A (pp) KIF15	0.888	0.9		0.99	0.182
KIF20A (pp) DEPDC1	0.775			0.826	0.257
KIF20A (pp) KIF2C	0.639	0.9		0.967	0.159
KIF20A (pp) KIF23	0.79	0.9		0.983	0.28
KIF20A (pp) INCENP	0.469		0.143	0.883	0.763
KIF20A (pp) CEP55	0.951		0.16	0.973	0.394
KIF20A (pp) RACGAP1	0.781	0.9	0.62	0.995	0.554
KIF20A (pp) GTSE1	0.686			0.829	0.477
KIF20A (pp) KIF4A	0.873	0.9	0.112	0.991	0.359
MYL2 (pp) TPM2	0.186	0.9	0.111	0.955	0.46
DEPDC1B (pp) RHOB		0.9		0.9	
GCAT (pp) GATM		0.9		0.901	0.053
C15ORF23 (pp) CDK1	0.689			0.749	0.228
C15ORF23 (pp) NUF2	0.422			0.811	0.686
C15ORF23 (pp) CENPE	0.327		0.042	0.823	0.748
GTSE1 (pp) MKI67	0.67			0.721	0.189
GTSE1 (pp) CDK1	0.671	0.9		0.978	0.401
GTSE1 (pp) TPX2	0.801			0.829	0.179
GTSE1 (pp) BIRC5	0.738			0.764	0.136
GTSE1 (pp) CENPE	0.685			0.726	0.166
GTSE1 (pp) KIF2C	0.756			0.799	0.212
CEP97 (pp) CDK1	0.075	0.9	0.159	0.92	0.094
EPSTI1 (pp) TLR3	0.742			0.781	0.188
EPSTI1 (pp) GBP	0.784			0.784	
DHX58 (pp) TLR3	0.475			0.868	0.759
DHX58 (pp) GBP	0.701			0.734	0.147
DHX58 (pp) EPSTI1	0.795			0.795	
DHX58 (pp) RSAD2	0.707			0.912	0.714
DHX58 (pp) MX1	0.676			0.843	0.537
DHX58 (pp) OASL	0.764		0.362	0.908	0.443
DHX58 (pp) ISG12-2	0.784			0.854	0.355
DHX58 (pp) USP18	0.678			0.746	0.244
